# Microbial Diversity and Hydrocarbon-Oxidizing Bacteria in Coastal Waters and Sands Contaminated by the Fuel Oil Spill in the Black Sea

**DOI:** 10.3390/microorganisms14081856

**Published:** 2026-08-20

**Authors:** Ekaterina M. Semenova, Alexey P. Ershov, Tamara L. Babich, Diyana S. Sokolova, Nataliya G. Loiko, Elena A. Bakay, Ekaterina S. Kazak, Tamara N. Nazina

**Affiliations:** 1 Winogradsky Institute of Microbiology, Research Center of Biotechnology of the Russian Academy of Sciences, 119071 Moscow, Russia; semenova_inmi@mail.ru (E.M.S.); e.alexey.mail@yandex.ru (A.P.E.); microb101@yandex.ru (T.L.B.); sokolovadiyana@gmail.com (D.S.S.); loikonat@mail.ru (N.G.L.); 2 Faculty of Geology, Lomonosov Moscow State University, 119234 Moscow, Russia; bakay_lena@inbox.ru (E.A.B.); kanigu@mail.ru (E.S.K.)

**Keywords:** Black Sea, fuel oil spill, hydrocarbon-oxidizing bacteria, bioremediation, 16S rRNA gene sequencing, prokaryotic community composition, natural attenuation, Kerch Strait accident

## Abstract

In 2024, an accident involving two tankers in the Kerch Strait resulted in the release of approximately 2400 tons of fuel oil into the Black Sea, causing significant contamination of seawater and the coastal zone. This study presents the first microbiological and molecular–ecological assessment of prokaryotic community composition and hydrocarbon-oxidizing bacteria (HOB) in coastal seawater and sand near Anapa (Russian Federation) following the spill. The taxonomic composition of nine samples was analyzed using high-throughput sequencing of 16S rRNA genes (V3–V4 regions), identifying *Bacteria* as the dominant domain (85.3–99.8%). In seawater samples, bacteria of the phyla *Pseudomonadota*, *Cyanobacteriota*, and *Bacteroidota* and archaea of the phyla *Thermoplasmatota* and *Crenarchaeota* predominated. Eighteen aerobic bacterial strains, including members of the genera *Shewanella*, *Pseudoalteromonas*, *Halopseudomonas*, *Marinomonas*, *Pseudomonas*, *Vibrio*, *Alcanivorax*, *Ectopseudomonas*, *Nitratireductor*, and *Echinicola,* were isolated from the zone of fuel oil spill. Several isolates demonstrated heavy oil degradation and biosurfactant production. Screening of collection strains isolated from other habitats revealed that *Rhodococcus erythropolis* TG65 and *Marinobacter lutaoensis* Pd1 and Pd2 degraded 92–94% of fuel oil *n*-alkanes. Elevated dissolved iron concentrations in the seawater indicate the possibility of a metabolic coupling between hydrocarbon oxidation and microbial iron reduction, mediated by indigenous *Shewanella* and *Pseudomonas* species. These findings indicate that indigenous HOB may contribute to the natural attenuation of aliphatic hydrocarbons in fuel oil.

## 1. Introduction

The widespread use of oil and petroleum products in the global economy necessitates their transport from production and refining areas to consumers. Anthropogenic activities and natural oil seeps result in a release between 4.7 and 83 million tons of oil into the marine environment annually [1]. Globally, there have been several major accidents involving tankers carrying oil and fuel oil, as well as the destruction of offshore production wells, accompanied by oil pollution of seas and environmental problems. These include accidents in the Gulf of Mexico (1980 and 2010), the Persian Gulf (1983 and 1991), the Mediterranean Sea (1991, 1998), Brittany (1978), Alaska (1989), Galicia (Spain, 2002), and others [2,3,4,5,6]. These accidents resulted in the release of tens to hundreds of thousands of tons of oil or fuel oil into the marine environment. The extent and duration of the impact of hydrocarbons on the marine ecosystem are determined by the quantity, composition, density, and viscosity of the spilled oil or fuel oil, as well as by current and wind speed and direction, temperature, and other environmental conditions.

Unlike higher organisms, whose growth is inhibited by various oil fractions, microorganisms are capable of degrading oil and make a significant contribution to the bioremediation of marine habitats [7]. The impact of oil on marine microbiota has been studied in the area of the world’s largest marine environmental pollution event, in the northern Gulf of Mexico. In 2010, approximately 200 million gallons of light Louisiana sweet crude oil entered the sea from BP’s Deepwater Horizon (DWH) exploratory well, located at a depth of 1500 m below the ocean surface [4,7,8,9,10,11]. In water samples from the dispersed oil plume, an increase in indigenous *γ-Proteobacteria* was observed compared to non-plume samples. The detected *γ-Proteobacteria* belonged to the order *Oceanospirillales* and comprised known psychrophilic hydrocarbon degraders and microorganisms from hydrocarbon-dominated environments, including *Oleispira antarctica*, *Thalassolituus oleivorans*, and *Oleiphilus messinensis* [4]. Also, the microbial incubations obtained from DWH discharge zone were enriched in *Oceanospirillum*, *Cycloclasticus*, *Colwellia*, *Pseudoalteromonas*, methylotrophic bacteria of the genus *Methylophaga*, and bacteria of the order *Rhodobacterales* [12,13]. The high rates of microbial methane oxidation recorded in the gas-rich deep-sea plume in the Gulf of Mexico at the beginning of the accident decreased significantly after a few months due to nutrient or oxygen depletion [14]. Using stable-isotope probing and single-cell genomics, it was suggested that bacteria of the genus *Colwellia* likely participate in the oxidation of ethane and propane, *Oceanospirillum* spp. oxidize cyclohexane, and *Cycloclasticus* spp. degrade polycyclic aromatic hydrocarbons [12,13,14,15]. Subsequent studies showed that over time, the oil underwent microbial degradation, the abundance of microorganisms increased, and the composition of the microbial community in the oil plume zone changed [10]. In this zone, bacteria of the order *Oceanospirillales* predominated, including psychrophilic and psychrotolerant species observed in low-temperature marine environments, such as *Spongiispira norvegica* and *Oceaniserpentilla haliotidis* (previously referred to in DWH as *Oceanospirillum* [4,13]). The presence of aerobic microorganisms and functional genes for aerobic oil degradation was consistent among all water samples in the DWH oil spill zone, whereas a greater number of *δ*-*Proteobacteria* and functional genes associated with anaerobic oil degradation were found in sediments located closest to the DWH discharge site [9].

In 1989, as a result of the accident involving the oil tanker Exxon Valdez on Bligh Reef in Prince William Sound, Kentucky (USA), 38,000 tons of heavy crude oil entered the sea, leading to contamination of the marine environment and 2000 km of shoreline in Prince William Sound and the Gulf of Alaska [16]. The API gravity of the light Louisiana crude oil released in the Gulf of Mexico (Macondo oil) was 35.2, whereas that of the heavy oil from the Exxon Valdez tanker in Alaska was 29.0. In 1989, Bragg et al. [17] estimated the background rates of oil biodegradation at 1.3 g oil/(kg sediment/yr) for surface oil and 0.8 g oil/(kg sediment/yr) for subsurface oil. The numbers of oil-degrading bacteria during this period were (1–5) × 10^3^ cells/mL of seawater, or approximately 1–10% of the total heterotrophic bacterial population. In late 1989, the numbers of oil-degrading bacteria increased to approximately 1 × 10^5^ cells/mL, which constituted about 40% of the heterotrophic population in oiled shoreline pore waters. It was concluded that indigenous oil-degrading bacteria played a significant role in reducing the environmental impact of both the Exxon Valdez and BP Deepwater Horizon oil spills [7]. Twenty and twenty-five years after the accident, residues of the spilled oil from the tanker vessel Exxon Valdez are still found within the beaches of Prince William Sound [18,19]. The oil was only slightly weathered and contained high concentrations of polycyclic aromatic hydrocarbons (PAHs) harmful to the marine and coastal ecosystem [20,21]. Background concentrations of nutrients (nitrogen and phosphorus), dissolved oxygen (DO), and salinity were lower than optimal values for oil biodegradation, limiting aerobic oil biodegradation [18]. However, the presence of nitrate at low concentrations in the oiled pits did not rule out anaerobic oil degradation under anoxic conditions via denitrification. Using real-time data, numerical modeling, and scenario analysis, Atodiresei and co-workers [6] simulated different oil spill evolution scenarios. Using a medium density oil as an example, they calculated that 27% of light oil fractions reach the water surface and evaporate completely within a short period, whereas 72% persist in the marine environment and pose a higher risk of coastal pollution.

In November 2002, the sinking of the tanker Prestige, carrying 77,000 tons of high-sulfur fuel oil, caused the largest environmental catastrophe in Europe in the Bay of Biscay off the coast of Galicia, Spain, causing significant damage to the flora and fauna of the region [7,22,23].

Compared to crude oil pollution, contamination of the marine environment by fuel oil (or mazut) represents a more complex environmental problem [23,24,25]. Fuel oil comprises a complex mixture of heavy components remaining after the distillation of light fractions of crude oil (gasoline, kerosene, and diesel), has a higher specific gravity than crude oil and water, solidifies at 25 °C, and remains suspended at varying depths or settles to the bottom of the water body. The heavy fractions of fuel oil, particularly the resin and asphaltene fractions, are difficult to degrade and can be transported by currents or persist on the seabed for decades, whereas light fractions (gasoline, which is used to reduce the viscosity of fuel oil) reach the water surface and evaporate completely within a short period [25,26,27].

A significant part of Russian oil is transported using sea tankers. In the Kerch Strait, which connects the Azov and Black Seas, accidents involving tankers transporting fuel oil have occurred repeatedly [28,29,30]. On 15 December 2024, as a result of an accident involving two tankers, approximately 2400 tons of fuel oil entered the sea. Strong currents from the Azov Sea side led to the greatest contamination of the Black Sea zone adjacent to the strait. After the accident, a qualitative and quantitative analysis of hydrocarbons in the area of fuel oil contamination as well as rheological properties of fuel oil released were performed [30,31,32], while no microbiological studies were conducted.

Soloveva et al. [31] showed that three months after the accident, hydrocarbon concentrations in the surface water of the Kerch Strait area ranged from 0.01 to 0.27 mg·L^−1^, with the maximum permissible concentration (MPC, 0.05 mg·L^−1^) being exceeded at 6 out of 13 stations. These authors suggest that the distribution of *n*-alkanes and the composition of hydrocarbons indicate actively ongoing self-purification processes; however, information on microorganisms capable of participating in the degradation of petroleum products in the fuel oil spill zone is still lacking. Rapid transformation of petroleum aliphatic hydrocarbons in suspended matter from surface waters and in the surface layer of bottom sediments in the Kerch Strait has also been noted by other authors [29,30,31,32].

The aim of the present work was to determine the diversity of prokaryotes in coastal seawater and sand in the fuel oil spill zone of the Black Sea near Anapa using a molecular 16S rRNA gene-based approach, as well as to assess the abundance and isolate aerobic hydrocarbon-oxidizing bacteria potentially responsible for the self-purification capacity of the marine environment.

## 2. Materials and Methods

### 2.1. Research Subjects and Sampling

**Figure 1:**
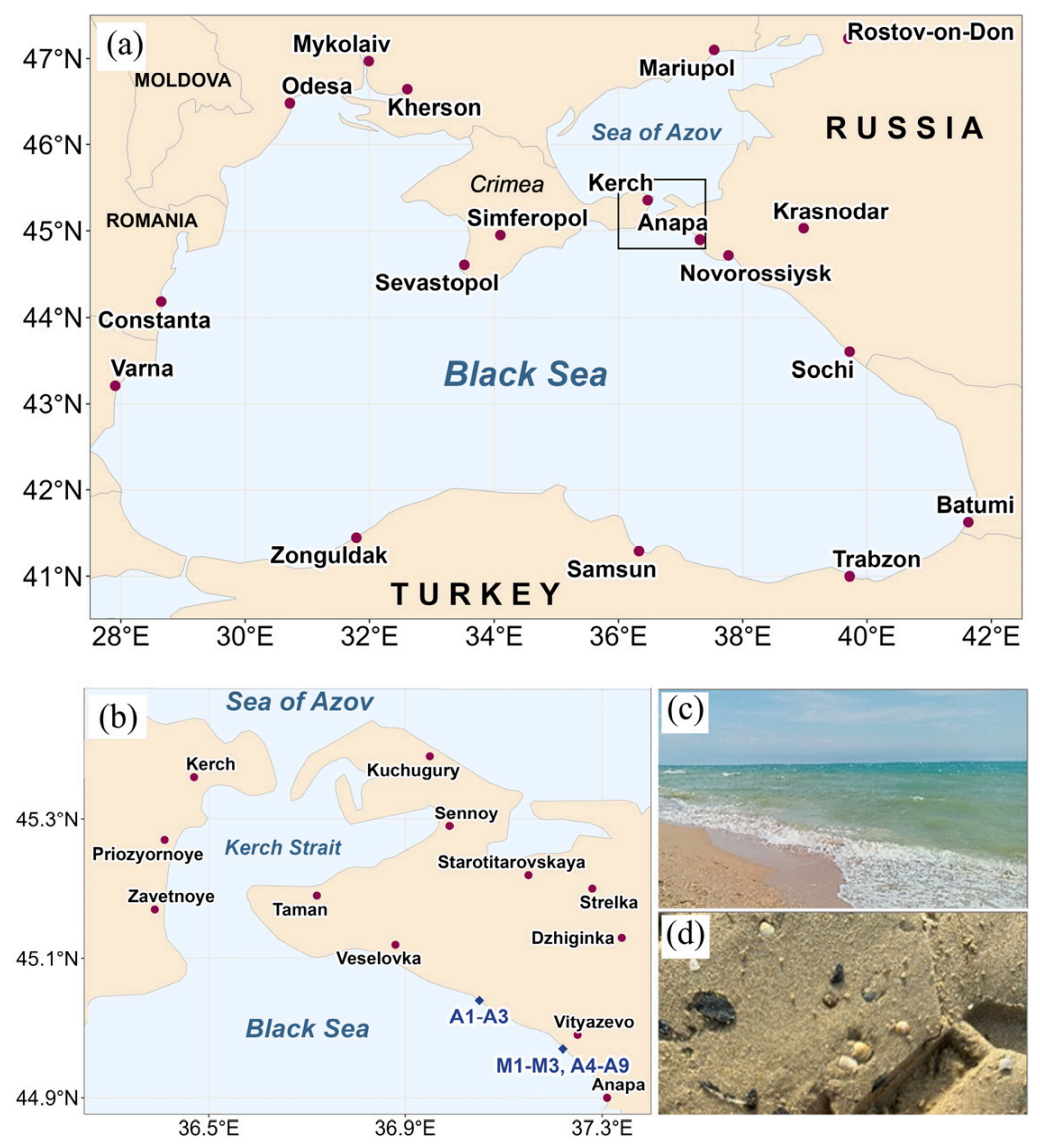
Map showing the location of the sampling sites (**a**,**b**) and general views of fuel oil-contaminated seawater (**c**) and sand (**d**) in the Black Sea near Anapa (Krasnodar Krai, Russia). The sampling sites are marked with blue squares.

Samples of seawater, sand, and fuel oil were collected near Anapa (Krasnodar Krai, Russian Federation) following the accident involving tankers transporting fuel oil that occurred on 15 December 2024 in the Kerch Strait, which connects the Azov and Black Seas (Figure 1). The list of samples collected from the fuel oil contamination zone is provided in Table 1.

The first sampling of seawater, contaminated sand, and a fuel oil pellet from the water surface (samples A1, A2, and A3, respectively) was carried out on 22 January 2025, in the area of Zhara Beach near Blagoveshchenskaya station (Krasnodar Krai, Russian Federation) (45.05577° N, 37.07180° E). The second sampling of another set of seawater (A4 and A8) and contaminated sand (A5–A7 and A9) was carried out on 3–4 June 2025, on the beaches of the village of Vityazevo (Krasnodar Krai, Russian Federation) (44.977993° N, 37.255162° E). In January 2025, the air temperature in the Anapa area ranged from +1 to +7 °C, and the average water temperature was 10 °C [https://world-weather.ru/pogoda/russia/anapa/water-january, accessed on 1 July 2026] [33].

In June 2025, the air and water temperatures were +15 … +25 °C and +22 °C, respectively [https://world-weather.ru/pogoda/russia/anapa/water-june/, accessed on 1 July 2026] [34]. Seawater samples were taken into sterile bottles near the shore from a depth of 0.3 m. Sand and fuel oil were collected into sterile Falcon tubes. Seawater samples A1, A4, and A8, for microbial community analysis, fixed with ethanol (70:30, *v*/*v*), were filtered through membrane filters with a pore size of 0.22 µm (Millipore, Burlington, MA, USA) and stored at −20 °C. Samples for microbiological analysis were stored for 2 days at 6 °C until analysis.

In addition, the composition of three fuel oil samples M1–M3 (containers with fuel oil, fuel oil with water, and fuel oil with sand in layers) collected from the beach of Anapa and obtained on 24 December 2024, from Professor I.V. Perminova (Department of Chemistry, Moscow State University, Moscow, Russia) was investigated. It is likely that the heavy fraction of the fuel oil remained in the tankers and settled to the bottom. At the same time, the films on the water surface and the fuel oil washed ashore represented its light fraction, possibly corresponding to the diesel fraction added to fuel oil to lower its pour point.

### 2.2. Analytical Methods

#### 2.2.1. Physicochemical Analyses of Seawater and Tensiometry

The content of major ions and selected trace elements in the seawater samples was determined by capillary electrophoresis using a Capel-205 system (Lumex, Saint Petersburg, Russia). Trace element concentrations (Li, Be, Al, Ti, V, Cr, Mn, Co, Ni, Cu, Zn, Rb, Zr, Mo, Cd, Sn, Cs, B, W, Pb) were measured using high-resolution inductively coupled plasma mass spectrometry (HR-ICP-MS) on an ELEMENT 2 instrument (Thermo Fisher Scientific, Waltham, MA, USA).

The surface tension of the culture liquid at the air interface and the interfacial tension at the phase boundary between the culture liquid and hexadecane were determined in three replicates using a DST 60 tensiometer (Surface Electro Optics, Suwon, Republic of Korea) by the Du Noüy ring method using a platinum ring. Prior to interfacial tension measurement, samples with 15 mL of hexadecane were kept for 20 min at 25 °C to obtain a clear phase boundary.

#### 2.2.2. Hydrocarbon Analysis

Extraction and fractionation of oil and fuel oil. To cultures grown on crude oil or fuel oil, 25 mL of *n*-hexane was added per 150 mL of culture liquid. The mixture was vigorously stirred, and the non-polar fraction was extracted from the mixture for 10 min (25 °C). An aliquot of 10 mL of the non-polar fraction containing dissolved oil components was transferred to a separation column containing 3 g of silica gel as the sorbent. Hexane was used as the eluent. After passing through the column, the sample was evaporated to 50 µL and then analyzed by gas chromatography.

Gas chromatography of oil alkanes. The alkane content in oil degraded by microorganisms was determined in three replicates in comparison with a sterile control sample. One microliter of the paraffin fraction of the oil, separated according to the method described above, was analyzed on a Kristall 5000.1 gas chromatograph (SKB Khromatek JSC, Yoshkar-Ola, Russian Federation) equipped with a flame ionization detector at 320 °C, using a ZB-1 capillary column over a temperature range of 100–310 °C. Helium was used as the carrier gas. The residual *n*-alkane content was calculated as the ratio of the sum of *n*-alkanes to the sum of *iso*-alkanes in the sample, divided by the same ratio in the control sample with non-degraded oil (the ratio in the control was taken as 100%).

Determination of Total Petroleum Hydrocarbon (TPH) content. The loss of TPH content in the culture liquid was assessed in three replicates by extraction-photometric method [35] using an AN-2 oil products analyzer (Neftekhimavtomatika, St. Petersburg, Russian Federation). Non-polar oil compounds were extracted from 150 mL of culture liquid by adding 30 mL of tetrachloroethylene (TCE) and vigorous stirring. The oil products were then separated from polar hydrocarbons on a column filled with aluminum oxide. Subsequently, 0.2 mL of the extract was taken, diluted with 1.9 mL of TCE, and the hydrocarbon content was measured photometrically in the infrared region at a fixed wavelength of 540 nm according to the instrument manufacturer’s recommendations.

#### 2.2.3. Fuel Oil Analysis

Fuel oil consists of the following analytical groups (according to Russian terminology): asphaltenes, resins (acidic and neutral), and oils. This separation is approximately analogous to SARA (saturates, aromatics, resins, asphaltenes) [36]. Asphaltenes were separated by filtering fuel oil diluted forty-fold with *n*-hexane through filter paper. The deasphalted fuel oil was then separated on a liquid chromatography column. The column contained silica gel in equilibrium with a solvent. The solvents used sequentially for the mobile liquid phase were as follows: *n*-hexane (C_6_H_14_) for oils, benzene (C_6_H_6_) for neutral resins, and alcohol-benzene (1:1) for acidic resins. Saturated and aromatic hydrocarbons were extracted from deasphalted fuel oil with *n*-hexane (96%) and toluene, respectively, in a glass column packed with AgNO_3_-impregnated silica gel. Fractions were analyzed using an Agilent 7890B/5977A series Gas Chromatograph/Mass Selective Detector (Agilent Technologies, Santa Clara, CA, USA) with MS detection.

Hydrocarbon separation was carried out on a 60 m (i.d. 0.25 mm) quartz capillary column SGE-1ms (0.25 µm film thickness) under linear temperature programming from 50 °C to 320 °C at a rate of 3 °C·min^−1^. Helium was used as the carrier gas. Compound identification and processing of the GC-MS data were performed using Xcalibur software v. 2.1 (Thermo Electron Corporation, Waltham, MA, USA).

### 2.3. Media Composition and Isolation of Aerobic Bacteria

The number of aerobic bacteria was determined via the Most Probable Number (MPN) method by inoculating 10-fold dilutions of seawater samples into liquid media containing various substrates. Microbial counts in sand samples were performed as follows: a representative sample (10 g) of each collected sand sample was added to 90 mL of sterile water and mixed in a rotary shaker at 250 rpm and 23 °C for 10 min according to standard procedures for soil samples. An aliquot (1 mL) of the supernatant suspension was used as inoculum for MPN estimations, and 10 mL of each suspension was filtered through membrane filters with a pore size of 0.22 µm (Millipore, Burlington, MA, USA) and stored at −20 °C for molecular studies.

For enumeration of bacteria, a liquid mineral medium was used containing the following components per liter of distilled water: 5.4 g MgCl_2_·6H_2_O, 4.0 g Na_2_SO_4_, 1.5 g CaCl_2_·H_2_O, 0.2 g NaHCO_3_, 0.2 g Na_2_HPO_4_, 0.5 g NH_4_Cl, 0.68 g KCl, 0.1 g KBr, 0.025 g H_3_BO_3_, 0.024 g SrCl_2_·6H_2_O, 0.002 g NaF, 18.0 g NaCl; pH 7.2 ± 0.2. The number of aerobic organotrophic bacteria (AOB) was determined on mineral medium supplemented with 1.0 g/L yeast extract, 2.0 g·L^−1^ tryptone, and 2.0 g·L^−1^ glucose.

The number of aerobic hydrocarbon-oxidizing bacteria was estimated in liquid mineral medium with 0.1% (*v*/*v*) sterile fuel oil injected directly into the vial, in stationary conditions. Liquid incubations were shaken gently once a week to provide more uniform spreading of hydrocarbons in the flasks. Hydrocarbon-oxidizing bacteria were also incubated on solid mineral medium containing 2% (*w*/*v*) agar and fuel oil (0.1%, *v*/*v*) smeared on the surface of the solid medium (agar plate) right before the inoculation. The MPN experiments were carried out in triplicate. Taking into account the average values of sea water temperatures in January and June 2025 (+10 and +22 °C, respectively [33,34]), enrichments were incubated at 15 °C and 23 °C for 14–30 days.

Pure cultures were isolated by sequential subculturing from liquid to solid medium containing 0.1% (*v*/*v*) diesel fuel or fuel oil. Several strains were isolated by direct inoculation of fuel oil-contaminated sand onto solid marine medium without substrate addition. Pure cultures were maintained on a solid medium used for enumeration of AOB, taking into account the optimal salinity for each strain. The purity of the strains was checked by colony microscopy and by sequencing of the 16S rRNA genes as described previously [37]. Scanning electron micrographs of total cells were obtained using a scanning electron microscope (Quattro S, Thermo Fisher Scientific Brno s.r.o., Brno-Černovice, Czech Republic) at an accelerating voltage of 15.0 kV as described by Semenova et al. [37].

### 2.4. DNA Isolation and Sequencing of the 16S rRNA Gene V3–V4 Fragments

Microbial biomass from seawater, fuel oil, and sand samples was washed off from filters with lysis solution (0.15 M NaCl and 0.1 M Na_2_EDTA, pH 8.0) and used for DNA extraction with the DNeasy PowerSoil Pro kit (Qiagen, Hilden, Germany). The DNA template was used for PCR amplification of the V3–V4 regions of the 16S rRNA genes using the primer pair 341F (5′-CCTAYGGGDBGCWSCAG-3′) and 806R (5′-GGACTACNVGGGTHTCTAAT-3′) [38]. The amplified V3–V4 fragments were sequenced using the MiSeq system (Illumina, San Diego, CA, USA) with a MiSeq Reagent Kit v3 (600 cycles) (Illumina, San Diego, CA, USA) as recommended by the manufacturer.

### 2.5. Sequencing Data Analysis

Raw reads from the Illumina platform were trimmed and demultiplexed, and then processed using the SILVAngs 1.4 online resource [39]. A standard amplicon analysis pipeline was performed, including alignment with SINA v. 1.2.10 [40], quality control [39], dereplication and clustering using VSEARCH v. 2.17.0 [41], and classification by BLASTn v. 2.11.0 [42] against the non-redundant SILVA SSU reference dataset v. 138.2 [39].

The resulting fingerprints were subsequently analyzed using R v. 4.5.2 with microeco package v. 1.16.0 [43,44]. The functional redundancy of the microbial communities was estimated with FAPROTAX database v. 1.2.10 [45,46]; LEfSe differential test [47] was performed to reveal significant biomarkers diverging the communities of seawater and sand; R packages ggplot2 v. 4.0.1 [48], ggrepel v. 0.9.6 [49], corrplot v. 0.95 [50], paletteer v. 1.6.0 [51], sf v. 1.1-0 [52,53], and rnaturalearth v. 1.2.0 [54] were used for visualization.

Microbial composition similarities were visualized in a Principal Coordinates Analysis (PCoA) ordination plot based on Bray–Curtis similarities of OTUs, using the R packages microeco and vegan v. 2.7-3 [55]. A statistical significance (*p*) of differences in bacterial community composition was assessed using permutational multivariate analysis of variance (PERMANOVA) in the vegan package in R. A significance level of α = 0.05 was considered for all statistical analyses.

### 2.6. Nucleotide Sequence Accession Numbers

The original sequencing data of the 16S rRNA gene V3–V4 fragments of prokaryotic communities presented in this study have been deposited at the National Center for Biotechnology Information (NCBI) Sequence Read Archive (SRA; available at http://www.ncbi.nlm.nih.gov/sra/ (accessed on 6 July 2026)) under accession number PRJNA1418990. The GenBank/EMBL/DDBJ accession numbers for the 16S rRNA gene sequences of the new bacterial strains are as follows: PX924859, PX924868, PX924912, PX924993, PX925089, PX931340, PX931356, PX931358–PX931360, PX931515, PX931626–PX931628, PX931687, PX931744.

## 3. Results and Discussion

### 3.1. Chemical Composition of Seawater

The studied seawater sample was slightly alkaline (pH 7.91), had a density of 1.012 g/cm^3^, and exhibited a sodium chloride composition, with a total dissolved solid (TDS) content of 18.46 g/L (Table S1). Overall, the macrocomponent composition and TDS of the studied seawater sample were comparable to those of pre-spill seawater reported previously [56].

The NH_4_^+^, NO_2_^−^, NO_3_^−^, and F^−^ contents were all below 2 mg/L. It should be noted that the content of strontium (Sr = 3.5 mg·L^−1^) and total iron (Feₜₒₜ = 3.16 mg·L^−1^) in the studied seawater sample exceeded the maximum permissible concentrations for fisheries according to Russian regulations (MPC Sr = 0.4 mg·L^−1^, MPC Feₜₒₜ = 0.1 mg·L^−1^) [57] by approximately 8.75-fold and 31.6-fold, respectively. The content of most trace elements did not exceed the MPCs, with the exception of lithium (Li), vanadium (V), nickel (Ni), copper (Cu), and zinc (Zn), which exceeded the MPCs by 1.2-fold, 22.9-fold, 1.5-fold, 6.4-fold, and 1.5-fold, respectively (Table S2). Previously, elevated concentrations of these trace elements exceeding MPCs were recorded in the surface and bottom waters of the Black Sea as a result of persistent anthropogenic pollution in the region or from submarine mud volcanoes in the Black Sea [58,59].

The elevated total iron concentration in the seawater sample relative to the MPC is noteworthy. As a redox-sensitive element, iron does not typically accumulate in dissolved form in near-surface marine waters, as it is highly unstable under oxidizing conditions and readily precipitates as iron (oxyhydr)oxides. However, the presence of an oil spill leads to the formation of a surface film, which likely inhibits the diffusion of atmospheric oxygen and promotes reducing conditions even in the upper seawater layer.

Oil pollution contributes to an increase in the concentration of dissolved organic matter (DOM) through hydrocarbon biodegradation in seawater. Magnetite (Fe_3_O_4_), hematite (α-Fe_2_O_3_), and iron hydroxides (FeOOHs) have previously been identified in suspended particulate matter and bottom sediments of the Black Sea [60]. The resulting Fe^2+^ forms stable complexes with DOM ligands, which inhibit its oxidation and promote its accumulation in dissolved form at elevated concentrations in seawater. Under natural conditions, this process typically occurs in bottom waters and sediments, which act as a geochemical barrier for iron [61]. In the present case, however, the oil spill acted as a catalyst for the accumulation of dissolved iron in the upper surface layers of seawater. However, this mechanism should be regarded as a hypothesis consistent with the observed geochemical conditions rather than as direct evidence from the present study. Its verification will require additional field observations and controlled experimental studies investigating the influence of oil contamination on iron cycling in marine environments.

The study by Soloveva et al. [31], conducted in March 2025, showed that the physicochemical parameters of the seawater remained within normal limits. Seawater temperature in the surface layer ranged from +8.5 to +10.0 °C. The pH values varied from 8.2 to 8.4, Eh varied from +85 to +134 mV, and the concentration of dissolved oxygen ranged from 9.6 to 10.9 mg·L^−1^; this characterizes the aquatic environment as an oxidizing one. The predominance of biodegraded hydrocarbons indicated that ongoing self-purification processes were actively occurring two months after the accident [31].

### 3.2. Composition of the Fuel Oil Samples

All three studied fuel oil samples were fluid at 20 °C and completely soluble in chloroform (CHCl_3_). The boiling point (first drop of condensed vapor) was 75 °C, and the yield of the fraction up to 95–100 °C was approximately 50%. The measured density of the fraction was 1.01 g·cm^−3^, which corresponds to that of Black Sea water. Apparently, the fuel oil samples from the containers represent an emulsion (fuel oil in water) or water in fuel oil (the result of reverse emulsification). Viscous oils with over 0.5% asphaltenes can form stable emulsions containing 30–80% water, resulting in a three- to four-fold increase in volume. Emulsions, commonly called mousse, slow several destructive processes and can persist in the marine environment for months [62].

Group composition and hydrocarbon composition of fuel oil. Upon separation of the fuel oil samples into analytical groups, the content of volatile components was 55–56% (Table S3). The asphaltene content in the samples was 6–7%. A predominance of acidic resins was characteristic, with their content being higher than that of neutral resins and higher than that of asphaltenes. A predominance of the naphthene-aromatic fraction over the methane-naphthene fraction was noted, with a saturate/aromatic ratio of 0.8.

The malthene fraction of the fuel oil displayed a complete series of normal alkanes from *n*-C_9_ to *n*-C_31_, with alkanes of composition *n*-C_17_–*n*-C_23_ present at maximum concentrations (Figure S1). Among the iso-alkanes (*i*-C_15_–*i*-C_20_), phytane (*i*-C_20_) was present at the maximum concentration, with an *i*-C_19_/*i*-C_20_ ratio of 0.51. A naphthenic background (unresolved complex mixture, UCM) was visible on the chromatogram, also noted by other researchers in fuel oil samples from the surface horizon of the Kerch Strait [31].

**Figure 2:**
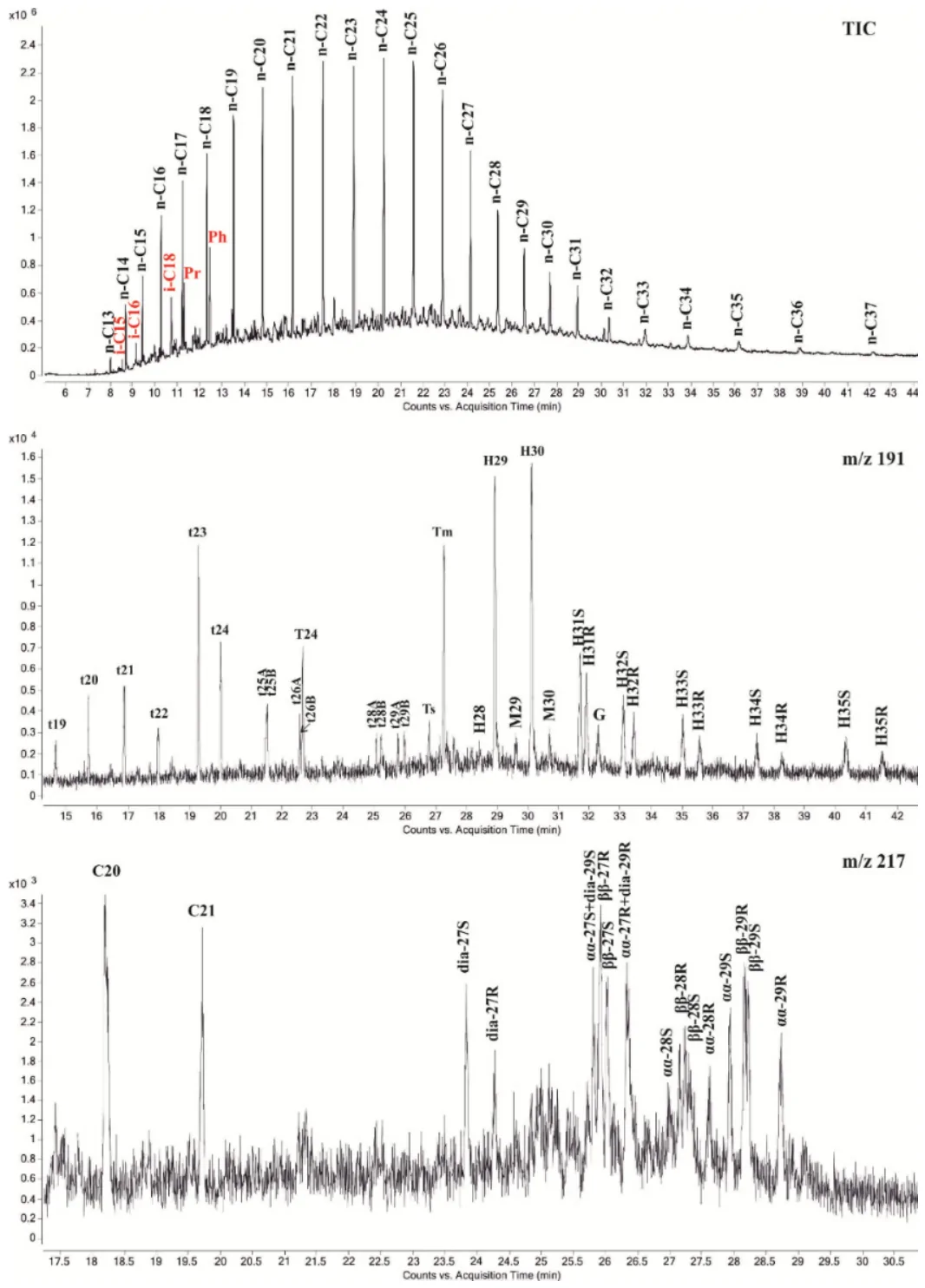
Total Ion Chromatogram (TIC) of saturated hydrocarbons and ion (*m*/*z* 191 and 217) chromatograms showing terpane and sterane distributions in the fuel oil.

In the methane–naphthene fraction, the alkane distribution maximum was shifted toward the higher molecular weight region, specifically to *n*-C_19_–*n*-C_26_ (Figure 2). High-molecular-weight alkanes up to *n*-C_37_ were detected, although the concentrations of *n*-C_32+_ alkanes were insignificant.

Overall, the distribution corresponds to that described by Nemirovskaya et al. [63]. The concentrations of cyclic compounds in the methane-naphthene fraction were two to three orders of magnitude lower than the alkane concentrations. The cyclic biomarkers resist biodegradation, making them ideal compounds for identifying the origin and fate of spilled oil. Commonly used parameters derived from saturated and aromatic biomarkers are nearly identical to those used in petroleum geochemistry (Table S4) for determining the source of the fuel oil spill and correlate with the results of Zimens et al. [64].

Figure S2 presents chromatograms of the main aromatic hydrocarbons. Although polycyclic aromatic hydrocarbons (PAHs) comprise only a few percent of typical crude oil, they are the most acutely toxic components and are associated with many chronic and carcinogenic effects in animals. PAHs are generally more resistant to biodegradation than many saturated biomarkers and tend to persist in contaminated water and sediments [62].

### 3.3. Prokaryotic Diversity and Functional Prediction

Using high-throughput sequencing of the 16S rRNA gene V3–V4 fragments, the composition of prokaryotes was determined in nine samples of seawater, fuel oil, and sand from the pollution zone. The obtained libraries contained from 10,334 to 16,160 sequences assigned to 4098–6960 observed OTUs. The Good’s coverage values ranged from 62% to 80%, indicating relatively low level of prokaryotic diversity capturing during the analysis. This may be a consequence of rapid microbial composition shift under massive hydrocarbon pollution and an increase in the proportion of uncultivated prokaryotes that are difficult to detect using the methods used. Recently, the presence of new, active, and uncultivated hydrocarbon-degrading microbes in the deepwater plume from the Deepwater Horizon (DWH) oil spill has been demonstrated using metagenomic approaches [65]. Microbial α-diversity, including observed richness and the Shannon and Simpson diversity indices, is provided in Table S5. The A1 and A4 libraries (seawater) and the A6 library (sand) were characterized by the highest number of OTUs and the highest Shannon diversity index values among the studied samples.

**Figure 3:**
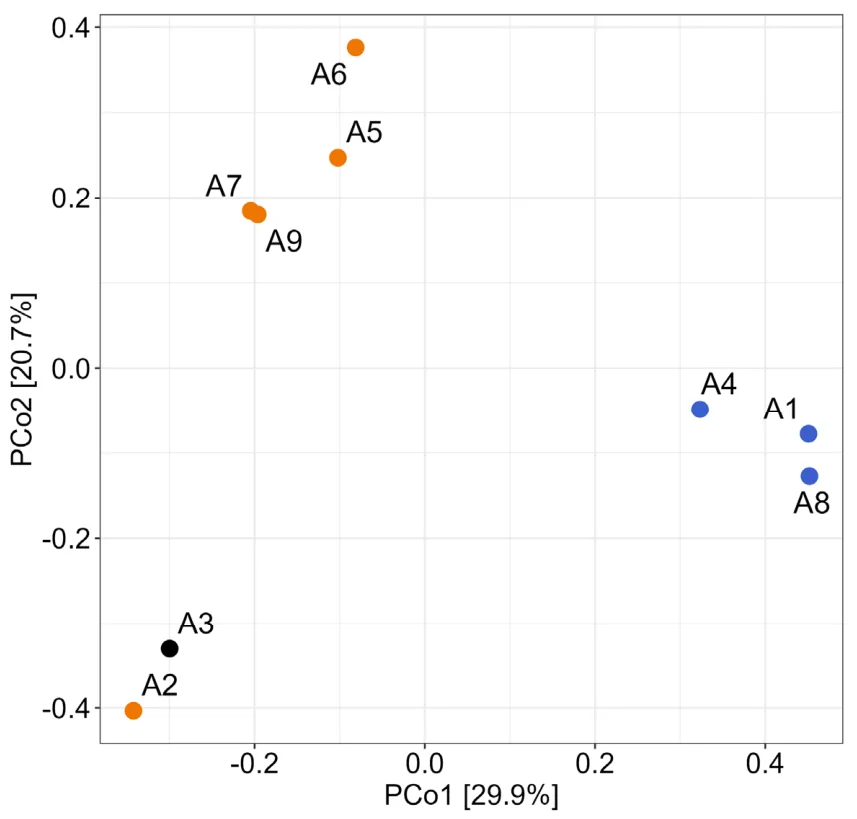
Principal coordinate analysis (PCoA) of microbial communities based on Bray–Curtis dissimilarity of 16S rRNA gene sequence libraries from the studied samples. Smaller distances between points indicate higher similarities between their bacterial communities. Samples of the same type—seawater, fuel oil, and sand—are represented by the same color: blue, black, and yellow, respectively.

To assess β-diversity of the microbial communities, a Principal Coordinates Analysis (PCoA) ordination plot based on Bray–Curtis similarity was performed (Figure S3). The studied microbial communities differed significantly across three groups (PERMANOVA, R^2^ = 0.887, *p* < 0.05), with homogeneous multivariate dispersions confirmed by PERMDISP (*p* = 0.068) (Figure 3 and Figure S3).

Despite differences in temperature and illumination of the seawater between the winter and summer periods, the component composition of the microbial communities in seawater samples A1, A4, and A8 was similar, and the communities clustered together, forming the first group. The second group comprised microbial communities from contaminated sand samples collected in June 2025 (A5–A7 and A9), which were similar in composition. The third group consisted of microbial communities from fuel oil-contaminated coastal sand (A2) and from the fuel oil itself (A3), collected one month after the accident, in January 2025.

**Figure 4:**
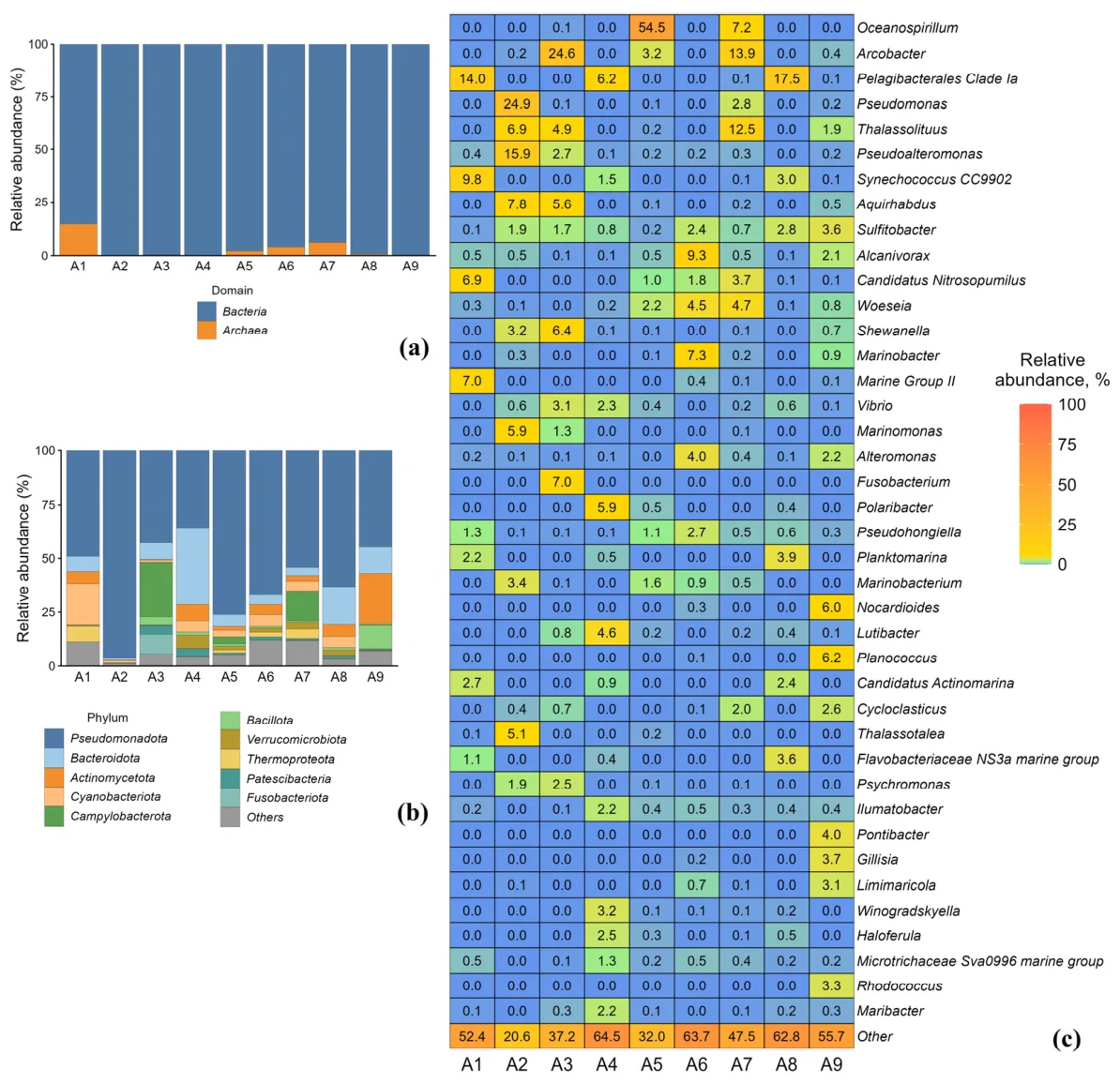
Taxonomic classification of prokaryotes at the domain (**a**), phylum (**b**) and genus level (**c**) based on 16S rRNA gene V3–V4 fragment sequences in libraries from seawater (A1, A4, and A8), sand (A2, A5–A7, and A9), and fuel oil (A3) samples.

Taxonomic characterization of microbial communities based on sequencing of V3–V4 fragments of the 16S rRNA gene and comparison with the SILVAngs 1.4 online database revealed prokaryotes affiliated in total with 64 phyla, 123 classes, 274 orders, 385 families, and 960 genus-level taxa (Figure 4a–c and Figure S4a–c). At the domain level, *Bacteria* were predominant, accounting for 85.3% to 99.8% of total sequences in the libraries. The highest content of *Archaea* was found in seawater sample A1 (14.7%) and in sand sample A7 (6.1%). Archaea of the genus *Candidatus* Nitrosopumilus from the phylum *Thermoproteota* (previously affiliated with the phylum *Thaumarchaeota*), known for their ability to oxidize ammonium [66,67], together with archaea from the phylum *Thermoplasmatota* (order Marine Group II), were mainly identified in the studied microbial communities. Archaea from the phylum *Nanoarchaeota* (order *Woesearchaeales*) and methanogens from the phylum *Halobacteriota* were detected in lower abundance (Figure S4a–c).

Bacterial profiling at the phylum level revealed *Pseudomonadota* (42.7–96.3%), *Bacteroidota* (1.0–17.5%), *Actinomycetota* (0.2–23.2%), *Cyanobacteriota* (0.6–19.4%), *Campylobacterota* (0.01–25.4%), *Bacillota* (0.06–10.7%), and *Thermoproteota* (0–7.1%) in the studied communities (Figure 4b and Figure S4a–c).

Genus abundance varied significantly among the studied samples, reflecting microbial heterogeneity (Figure 4c). In seawater sample A1, collected in winter, one month after the accident, typical marine bacteria predominated, including *Pelagibacterales* Clade Ia, *Planktomarina*, the cyanobacterium *Synechococcus*, as well as the archaea *Candidatus* Nitrosopumilus and Marine Group II (MGII) (*Candidatus* Poseidoniales, *Candidatus* Thalassarchaeaceae). *Ca.* Pelagibacterales, constituting 6.2–17.5% of the studied seawater samples, are common inhabitants of nearly every pelagic marine bacterioplankton community, as demonstrated by 16S rRNA gene analysis and metagenomic studies [68,69,70]. Bacteria of the genus *Planktomarina*, also detected in seawater (0.5–3.9%), belong to the globally distributed RCA cluster of the marine *Roseobacter* clade [71,72]. MGII archaea are globally abundant in the photic zone, with lower abundance in the deep sea, and have a worldwide distribution including polar regions [73,74] (Figure 4c). In seawater samples A4 and A8, collected in summer, 5.5 months after the accident, in addition to the aforementioned bacterial groups, bacteria of the genera *Polaribacter*, *Maribacter*, *Pseudomonas*, *Alcanivorax*, and *Thalassolituus* were also present, which include known hydrocarbon degraders [75,76,77,78].

In fuel oil sample A3, collected from the water surface, as well as in fuel oil-contaminated sand sample A2, both collected in January 2025, bacteria of the genera *Pseudomonas*, *Pseudoalteromonas*, *Aquirhabdus*, *Thalassolituus*, *Thalassotalea*, *Marinomonas*, *Marinobacterium*, and *Shewanella* were present, all known for their ability to degrade hydrocarbons in saline environments at low temperatures [14]. In other fuel oil-contaminated sand samples, typical marine bacteria of the genera *Oceanospirillum*, *Arcobacter*, *Thalassolituus*, *Alcanivorax*, *Marinobacter*, and *Cycloclasticus* were present, members of which are capable of utilizing both alkanes and aromatic hydrocarbons [14].

*Alcanivorax* spp., frequently encountered in marine habitats, are able to degrade aliphatic hydrocarbons, the alkyl groups of *n*-alkylbenzenes and *n*-alkylcycloalkanes, and a range of branched alkanes including pristane (produced by some marine zooplankton) more effectively than other hydrocarbon-degrading bacteria, which allows *Alcanivorax* to predominate in oil-containing seawater [75,79,80,81,82,83,84,85]. *Cycloclasticus* spp. participate in the removal of aromatic hydrocarbons from oil spills in marine environments [86]. Bacteria of the genera *Alcanivorax* and *Cycloclasticus* have been found in oil-contaminated environments in Delaware (USA), in the Gulf of Mexico, and on the Sakondani Coast (Japan) after the *Nakhodka* tanker accident [87,88,89,90].

In our study, every one of the nine samples comes from the contamination zone. There is no reference seawater or sand from an unaffected coastline against which to benchmark “baseline” Black Sea coastal microbiota. Thus, our interpretation on the participation of marine isolates in natural attenuation of the environment from fuel oil rests on comparison with literature baselines on other oil spills rather than paired empirical controls.

**Figure 5:**
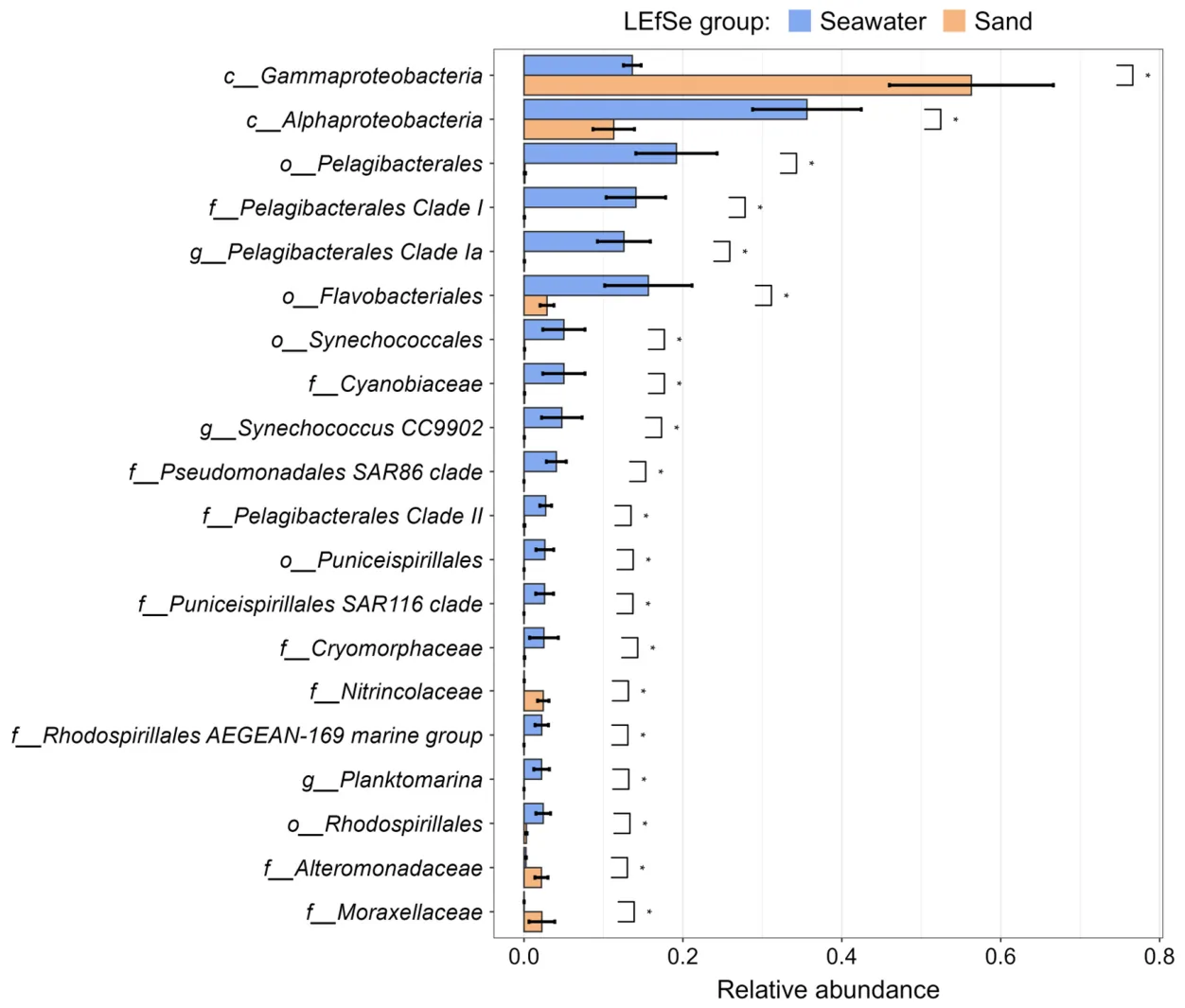
Relative abundances of the 20 most significant LEfSe biomarkers diverging the studied microbial communities depending on the sample type. Error bars represent standard errors; asterisks indicate significance of the differences.

To estimate differential abundance of the presented taxa, analysis of LEfSe (linear discriminant analysis effect size) was performed for two groups of sample type, seawater (16S rRNA gene libraries A1, A4, and A8) and sand (A2, A5–A7, and A9). Biomarkers were evaluated as significant if their log10 (LDA score) were 3.0 or higher. Analysis was performed on all taxonomy levels from phyla to genera. 95 taxa were selected after Kruskal–Wallis rank sum test at the first stage of LEfSe analysis. All biomarkers subsequently exceeded the LDA threshold are shown in the Figure S6. Relative abundances of the 20 most significant LEfSe biomarkers (Figure 5) clearly demonstrate predominance of *Alphaproteobacteria*, especially of *Pelagibacterales* Clade Ia and other representatives of this order, as well as *Synechococcus* and *Planktomarina* genera, in the seawater samples compared to the sand ones. In contrast, the samples of contaminated sand were characterized by the higher abundance of *Marinobacter* genus (*Alteromonadaceae* family) and other *Gammaproteobacteria*. The cladogram of the detected biomarkers is presented in the Figure S5. Thus, the significance of key taxa differential abundance was demonstrated with LEfSe analysis highlighting the possible shift in microbial community compositions under the hydrocarbon pollution.

Functional redundancy estimation using the FAPROTAX database v. 1.2.10 showed that aerobic and anaerobic chemoheterotrophy, fermentation, sulfate and sulfur respiration, nitrate reduction, oxygenic photoautotrophy, dark hydrogen oxidation, and hydrocarbon degradation were the main metabolic functions in the studied seawater and sand samples from the fuel oil contamination zone (Figure S7). Despite the constraints of the genus-based functional prediction, results of the analysis highlighted the shifts in presumable metabolic profiles of microbial communities in correspondence with the hydrocarbon pollution accident.

### 3.4. Isolation of Hydrocarbon-Oxidizing Bacteria from the Zone of Fuel Oil Spill

The abundance of culturable aerobic organotrophic bacteria in seawater samples (A1, A4, and A8), determined at 15 °C and 23 °C, ranged from 10^4^ to 10^5^ cells·mL^−1^, whereas the abundance of HOB was 1–3 orders of magnitude lower (Figure S8). In most of the studied sand samples, the abundance of aerobic organotrophs varied from 10^3^ to 10^9^ cells·g^−1^, and that of hydrocarbon-oxidizing bacteria ranged from 10^2^ to 10^8^ cells·g^−1^. The abundance of hydrocarbon-oxidizing bacteria in seawater was approximately similar in winter and summer samples, whereas the hydrocarbon-oxidizing population in coastal sand collected in June was more numerous than that in the winter sand sample.

**Figure 6:**
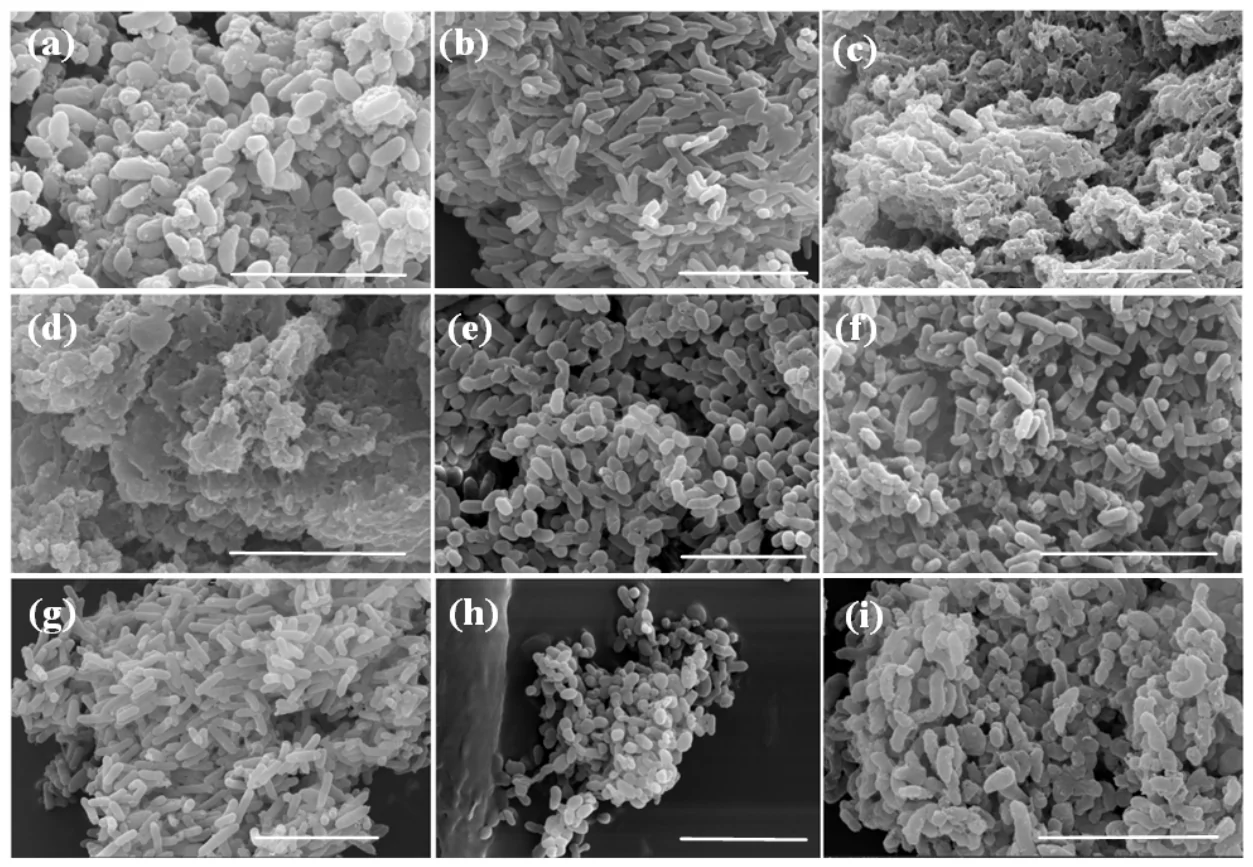
Scanning electron micrographs of cells of *Echinicola sediminis* Lo2 (**a**), *Ectopseudomonas khazarica* Nat2 (**b**), *Marinomonas foliarum* A24 (**c**), *Pseudoalteromonas arctica* A12 (**d**), *Pseudomonas kurunegalensis* A27 (**e**), *Pseudomonas peli* Lo4 (**f**), *Shewanella* sp. A22 (**g**), *Shewanella* sp. A14 (**h**), and *Vibrio diazotrophicus* A26 (**i**). The samples were examined under a scanning electron microscope (Quattro S, Thermo Fisher Scientific, Brno-Černovice, Czech Republic) at an accelerating voltage of 15 kV. Bars, 5 µm.

Eighteen aerobic bacterial strains were isolated from fuel oil-contaminated seawater, sand, and fuel oil itself, collected in January 2025. Cell morphology of several isolated strains is shown in Figure 6.

The strains were identified by 16S rRNA gene analysis. Taxonomic affiliation of the isolates is presented in the Table S6. The 16S rRNA gene similarity of most isolated strains to the genes of the type strains of the phylogenetically closest species was 99–100%, allowing the strains to be assigned to the corresponding species. 16S rRNA gene-based phylogenetic analysis showed that 16 isolated strains belonged to the class *Gammaproteobacteria* (genera *Shewanella*, *Pseudoalteromonas*, *Halopseudomonas*, *Marinomonas*, *Pseudomonas*, *Vibrio*, *Alcanivorax*, and *Ectopseudomonas*), one strain belonged to the class *Alphaproteobacteria* (genus *Nitratireductor*), and one strain belonged to the class *Cytophagia* (genus *Echinicola*). By direct inoculation of fuel oil-contaminated sand onto solid marine medium without substrate addition, the hydrocarbon-degrading strains *Alcanivorax dieselolei* Nat3, *Pseudomonas aeruginosa* Nat5, and *Ectopseudomonas khazarica* Nat2 and Lo5 were isolated. Strains isolated from coastal sand belonged to the genera *Pseudomonas*, *Vibrio*, *Marinomonas*, and *Ectopseudomonas.* From seawater, bacteria of the genera *Shewanella*, *Pseudoalteromonas,* and *Halopseudomonas* were isolated by sequential subculturing from liquid medium with hydrocarbons onto solid medium. However, the 16S rRNA gene similarity of strains *Shewanella* sp. A14, A15, and A22 to the genes of the phylogenetically closest type strains of this genus was 98.8–98.9%, slightly below the accepted threshold for assignment to a new species (98.7%) [91,92]. To clarify the taxonomic affiliation of these strains, genome sequencing and phylogenomic analysis are required, which was beyond the scope of the present study.

### 3.5. Growth of Marine Isolates on Heavy Crude Oil and Biosurfactant Production

The growth of marine isolates on heavy crude oil was tested (Table 2). The sterile control medium contained 1 mL of oil per liter (150 µL of oil per flask), and the alkane content in the oil was taken as 100%. The surface tension (ST) of the control medium against air was 70 ± 0.1 mN·m^−1^, and the interfacial tension (IFT) against hexadecane was 38.7 ± 1.3 mN·m^−1^. Strains *S. litoralis* A15, *E. khazarica* Nat2, and *P. peli* Lo4 degraded 46–68% of the added oil (Table 2). Oil degradation by most marine isolates was low, and for strains A11 and A16 it was within the margin of error, although slight alkane utilization was registered for strains A11, A12, A15, A17, and A22 (Figure S9). There is no contradiction here, since paraffin fraction in heavy crude oil constitute only a very small proportion of the total alkanes; therefore, even their complete consumption may be statistically undetectable.

Strains *E. khazarica* Nat2 and *P. peli* Lo4 demonstrated preferential utilization of paraffins from heavy crude oil (Figure S9e,j). Notably, the type strain TBZ2 of *Ectopseudomonas* (formerly *Pseudomonas*) *khazarica*, recently isolated from the Khazar (Caspian) Sea, geographically close to the Black Sea, was capable of degrading polycyclic aromatic hydrocarbons (naphthalene, phenanthrene, and anthracene) and grew in up to 8.5% NaCl (*w*/*v*) at 10–45 °C [93,94]. Bacteria of the species *P. peli* have been found to be capable of anaerobic U(VI) bioreduction in medium with acetate [95]. It is likely that the isolated strain *P. peli* Lo4, like many strains of the genus *Shewanella*, can carry out iron reduction, given the iron detected in the studied seawater sample [96]. However, the hypothetical possibility of iron (Fe^3+^) reduction by isolated marine strains in the presence of hydrocarbons should be tested experimentally.

Strains *P. arctica* A12 and *Shewanella* sp. A11 and A17 utilized 19–20% of the long-chain *n*-alkanes (primarily C_16_–C_25_) from heavy crude oil for growth; strains *H. gallaeciensis* A16 and *Shewanella* sp. A14, A15, and A22 degraded only 3–7% of the *n*-alkanes; and strains *P. kurunegalensis* A27, *M. foliarum* A24, *P. neustonica* A25, and *V. diazotrophicus* A26 hardly changed the fractional composition of the oil (Table 2).

Growth on crude oil of strains *H. gallaeciensis* A16, *P. arctica* A12, *P. kurunegalensis* A27, *Shewanella* sp. A14 and A15, *S. metallivivens* A17, *S. vesiculosa* A11, *V. diazotrophicus* A26, *E. khazarica* Nat2, and *P. peli* Lo4 was accompanied by the production of biosurfactants, resulting in a significant decrease in the surface tension (ST) of the culture liquid against air from 70 to 34–44 mN·m^−1^ and a decrease in the interfacial tension (IFT) against hexadecane from 39 to 0–2.4 mN·m^−1^ (Table 2). Strains *M. foliarum* A24, *P. neustonica* A25, and *Shewanella* sp. A22 slightly reduced surface and interfacial tension to 50–59 mN·m^−1^ and 16–39 mN·m^−1^, respectively.

Strain *Alcanivorax dieselolei* Nat3 was isolated from fuel oil-contaminated sand, and the fuel oil itself served as the primary growth substrate. The type strain *Alcanivorax dieselolei* B-5ᵀ was isolated from the surface water of the Bohai Sea (China), utilized C_8_ to C_28_ *n*-alkanes, and grew slowly on C_32_ and C_36_ *n*-alkanes [83]. When growing on C_24_ as the sole carbon source, strain B-5ᵀ produced biosurfactants that decreased the surface tension of the culture from 71.3 to 42.4 mN·m^−1^ after 7 days of cultivation.

### 3.6. Degradation of Fuel Oil Spill by Collection Hydrocarbon-Oxidizing Bacteria

**Figure 7:**
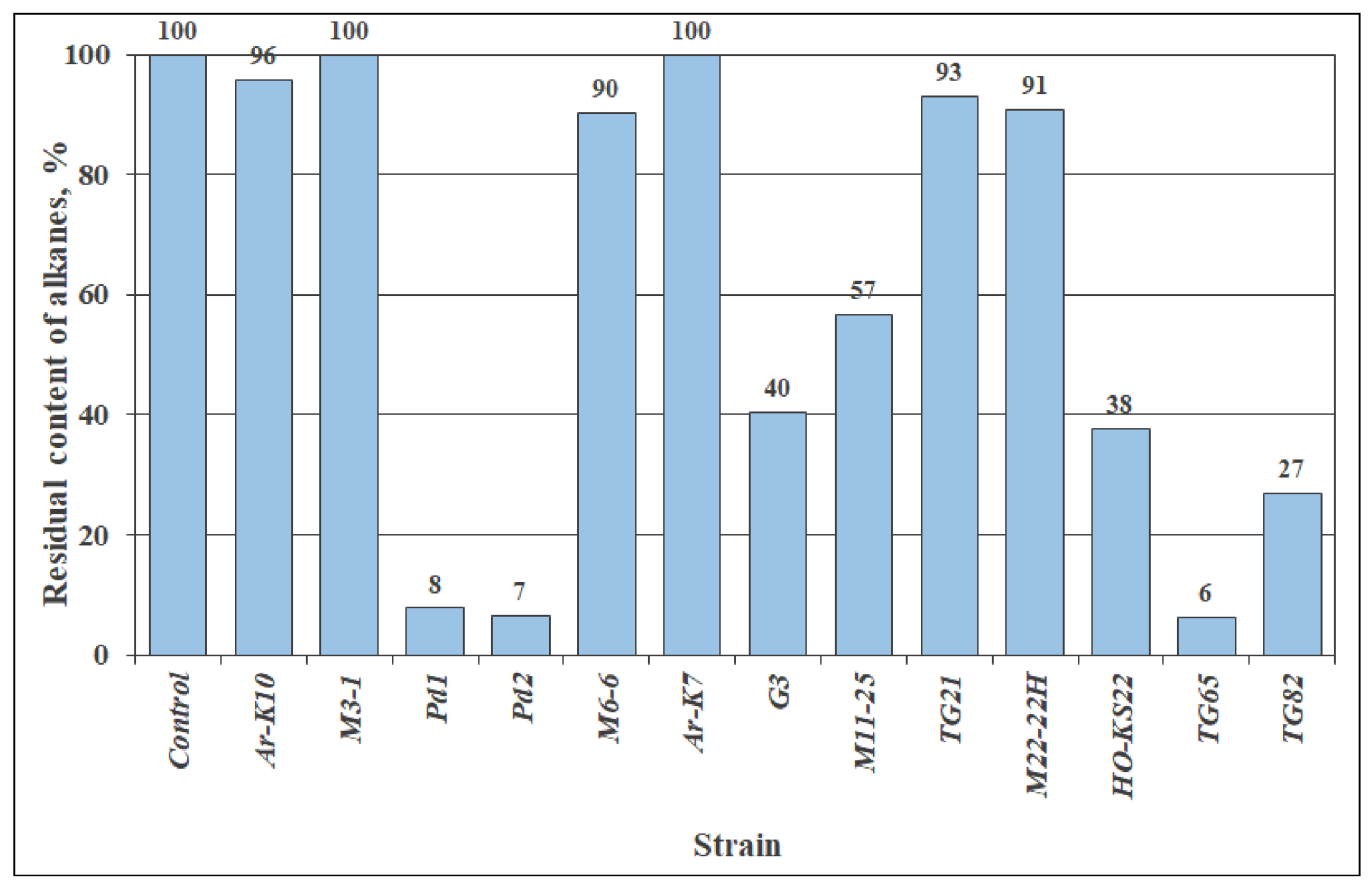
Residual content of *n*-alkane (in %) in fuel oil degraded by collection’s aerobic strains. Designations: Control, Control medium with fuel oil; Ar-K10, *Arthrobacter alpinus* Ar-K10; M3-1, *Arthrobacter salmonicida* M3-1; Pd1, *Marinobacter lutaoensis* Pd1; Pd2, *Marinobacter lutaoensis* Pd2; M6-6, *Pseudomonas brenneri* M6-6; Ar-K7, *Pseudomonas frederiksbergensis* Ar-K7; G3, *Ectopseudomonas guguanensis* G3; M11-25, *Pseudomonas kielensis* M11-25; TG21, *Pseudomonas kielensis* TG21; M22-22H, *Pseudomonas yamanorum* M22-22H; HO-KS22, *Rhodococcus erythropolis* HO-KS22; TG65, *Rhodococcus erythropolis* TG65; and TG82, *Rhodococcus erythropolis* TG82.

With the aim of identifying bacteria capable of degrading crude oil and fuel oil more effectively than the marine isolates described above, a screening was performed using 13 strains of hydrocarbon-oxidizing bacteria isolated from other habitats—petroleum reservoirs, oil-contaminated soil and water. These bacteria were obtained from the collection of the Laboratory of Petroleum Microbiology, Federal Research Center of Biotechnology, Russian Academy of Sciences. The taxonomic affiliation of the studied strains is indicated in Table S7. The degradation of fuel oil obtained from the accident site was assessed based on the total loss of petroleum products and on alkane loss, as demonstrated in the figures (Figure 7 and Figure S10).

Of the 13 strains tested, only 7 bacterial strains showed noticeable utilization of the *n*-alkanes present in the fuel oil. Strain *P. frederiksbergensis* Ar-K7 hardly utilized the alkane fraction of the fuel oil (Figure S10e). The best growth on fuel oil was shown by strains *Rhodococcus erythropolis* TG65 and *Marinobacter lutaoensis* Pd1 and Pd2, which degraded 92–94% of the *n*-alkanes. On the chromatograms of fuel oil degraded by strains *M. lutaoensis* Pd2 and *E. guguanensis* G3, a change in the baseline curve was visible, indicating a reduction not only in alkanes but also in other fuel oil components (Figure S10). Probably, of the tested collection strains, only *M. lutaoensis* Pd1 and Pd2, as well as *R. erythropolis* TG65, can be used in biotechnologies for bioremediation of surface ecosystems from fuel oil pollution.

### 3.7. Potential Applications of Isolated Strains in Bioremediation

Mechanical, physical and chemical methods are used to clean up hydrocarbon pollution, especially in the coastal marine zone; however, hydrocarbon-degrading microorganisms are ultimately responsible for the removal of spilled oil from deep-sea environments [2,5,7,10,97]. Bioremediation approaches include natural attenuation, biostimulation of indigenous microorganisms by supplying with exogenous nutrients and bioaugmentation based on adding natural or exogenous hydrocarbon degraders [3]. Understanding the influence of environmental conditions on the composition and efficiency of microbial communities decomposing hydrocarbons makes it possible to improve existing microbial-based remediation strategies.

The participation of a wide range of bacteria in the bioremediation of areas polluted with petroleum hydrocarbons at the Deepwater Horizon (DWH) drilling platform is shown [98,99]. The application of chemical dispersants in oil spills in order to break the oil film into small droplets and thereby increase the availability of hydrocarbons for microbes (that is, to stimulate biodegradation) does not always have a positive effect. In laboratory experiments were shown that the chemical Corexit dispersant, used after the Deepwater Horizon oil spill in the Gulf of Mexico, suppressed the growth of bacteria of the genus *Marinobacter*, which are effective natural destructors, and stimulated the growth of bacteria of the genus *Colwellia*, capable of degrading the components of the dispersant [100]. Limited effectiveness of bioremediation approach based on biostimulation with oleophilic fertilizers was revealed in the coastal area of Prestige heavy fuel oil spill [3], however application of oleophilic fertilizers in Alaska with the Exxon Valdez spill was effective [101].

It is likely that the formation of biosurfactants by natural hydrocarbon degraders will contribute to greater degradation of hydrocarbons in the area of oil spill.

The dominance bacteria of the phyla *Proteobacteria*, *Bacteroidetes*, and *Actinobacteria* have been registered in marine sediments of beaches from the northern Spanish coast affected by the Prestige oil spill [22,23,102,103].

The involvement of a wide range of bacteria has been shown in bioremediation of areas polluted with petroleum hydrocarbons at the Deepwater Horizon (DWH) drilling platform [9,10,11,12,99]. Bacteria of the genera *Alteromonas*, *Alcanivorax*, *Marinobacter*, *Thalassospira*, *Thalassobius*, *Labrenzia*, and *Bartonella* were the dominant members of the oil-degrading bacterial communities in seawater from the zone of the Deepwater Horizon (DWH) oil spill in the Gulf of Mexico [104]. *Alcanivorax*, *Marinobacter*, *Pseudomonas*, and *Acinetobacter* were detected in Gulf of Mexico Beach sands impacted by the deepwater horizon oil spill [105]. These bacteria are also found in unpolluted areas of the oceans [106]. It is likely that the supply of hydrocarbons from natural oil seepage and biogenic production of hydrocarbons by phytoplankton support the existence of a hydrocarbon-degrading population in the ocean [98,99,107,108,109]. However, in the oil spill zone, the proportion of these bacteria in the community increases significantly [110]. This is a biogeographic argument in favor of the existence of general patterns of the impact of oil pollution on the marine microbiota in the pollution zone and will allow us to predict this response in the future.

Hydrocarbon-degrading microbial communities identified in seawater and coastal sand in the area of the Black Sea oil spill, including *Alcanivorax*, *Marinobacter*, and *Shewanella,* were similar to assemblages found in the oil spill area of the Prestige tanker in Spain, the Nakhodka tanker in the Japan Sea, and Deepwater Horizon oil spill in the Gulf of Mexico [22,23,84,101,110,111].

Hydrocarbon-oxidizing bacteria isolated by the area of Black Sea fuel oil spill are physiologically adapted to marine habitat conditions and are capable of degrading individual components of heavy oil and fuel oil, making a definite contribution to natural attenuation. Strains *Pseudomonas peli* Lo4, *Ectopseudomonas khazarica* Nat2, *Shewanella vesiculosa* A11, and *Alcanivorax dieselolei* Nat3 were among marine isolates effectively degrading heavy oil and reducing the interfacial tension at the *n*-hexadecane interface. It is known that representatives of these genera are able to synthesize effective biosurfactants. Bacteria of the genus *Pseudomonas* synthesize rhamnolipids; *Bacillus* spp. produce surfactin; *Alcanivorax borkumensis* produce surface-active glycine-glucolipids; marine isolate *Shewanella algae* B12 produce glycolipid biosurfactant; and *Rhodococcus* synthesizes trehalose lipids [112,113,114,115,116].

It is likely that the new marine isolates from the area of Black Sea fuel oil spill can be used in surface bioreactors to remove hydrocarbons from emulsified spilled oil residues collected from seawater, or in floating booms in the form of a closed reservoir in the coastal zone.

### 3.8. Study Limitations

After the Kerch Strait accident, only twelve samples of fuel oil, seawater, and contaminated sands (six in winter and six in summer) were collected from five spatial points. Therefore, most of the nine sequenced microbial communities lacked biological replication, and the observed clustering in PCoA, PERMANOVA, and PERMDISP analyses were partly confounded by sampling source, season, and location, which reduces the statistical power of the tests performed and limits formal inference. Seasonal effects (temperature, salinity, nutrient availability, phytoplankton blooms) may influence microbial composition independent of hydrocarbon contamination. In LEfSe analysis, the samples within groups were also defined by spatial points rather than true biological replicates, i.e., the differences caused by the variation in sampling time and site were not considered. Given these constraints, the examination of microbial community composition in this work should be regarded as primarily exploratory and hypothesis-generating rather than strictly inferential. At the same time, the main phenomenological patterns are consistently supported by independent molecular data, and therefore this component of the study provides a valuable framework that complements and reinforces the subsequent physiological experiments.

## 4. Conclusions

The results of a study of the microbial community in the area of fuel oil pollution that occurred in 2024 in the Kerch Strait showed that seawater and coastal sediments in the Anapa contamination zone harbor a robust microbial community capable of aerobic degradation of alkanes, even under low-temperature conditions. Our findings suggest that these indigenous bacteria may be involved in the natural attenuation of fuel oil components within the marine ecosystem. Notably, the correlation between elevated iron concentrations and the presence of *Shewanella* and *Pseudomonas* supports the hypothesis of coupled alkanes and aromatic components of fuel oil degradation and iron reduction. Furthermore, the isolation of high-potential strains, including *Alcanivorax dieselolei* Nat3 and *Ectopseudomonas khazarica* Nat2, provides a promising basis for developing targeted biotechnological systems for the bioremediation of oil-polluted coastal soils. Future research will focus on the degradation of various components of fuel oil by the most promising marine isolates, the relationship between hydrocarbon oxidation and iron reduction, the taxonomic description of new strains, and obtainment of non-identified members of marine microbial communities.

## Figures and Tables

**Table 1:** List of seawater, coastal sediment, and fuel oil samples collected from the fuel oil contamination zone in the area of Anapa.

Sample ID	Sample Type	Location	Sampling Date	Coordinates
M1, M2, M3	Fuel oil	Anapa Beach	20 December 2024	44.977993° N, 37.255162° E
A1	Seawater	Zhara Beach, Blagoveshchenskaya	22 January 2025	45.05577° N, 37.07180° E
A2	Contaminated sand			
A3	Fuel oil pellet (surface water)			
A4	Seawater	Miracleon beach, Vityazevo	3 June 2025	44.977993° N, 37.255162° E
A5	Contaminated sand			
A6	Contaminated sand			
A7	Contaminated sand	Vityaz beach, Vityazevo	3 June 2025	44.977993° N, 37.255162° E
A8	Seawater			
A9	Contaminated sand	Greek beach, Vityazevo	4 June 2025	44.977993° N, 37.255162° E

**Table 2:** Biodegradation of heavy oil alkanes, and surface tension (ST) and the interfacial tension (IFT) against hexadecane of the culture liquid of the marine isolates. Data are presented as means ± SE (*n* = 3).

Genus, Species, Strain	ST, mN·m^−1^	IFT, mN·m^−1^	Residual Oil, µL·L^−1^	Residual Alkanes, %
Sterile control with oil	70.0 ± 0.1	38.7 ± 1.3	1000 ± 24	100 ± 0
*Shewanella vesiculosa* A11	43.8 ± 0.3	0.4 ± 0.2	984 ± 15	84 ± 4
*Halopseudomonas gallaeciensis* A16	39.1 ± 0.6	0.3 ± 0.1	960 ± 24	94 ± 6
*Vibrio diazotrophicus* A26	39.0 ± 1.8	1.3 ± 0.6	891 ± 20	100 ± 0
*Shewanella litoralis* A22	55.1 ± 1.1	30.5 ± 0.9	858 ± 20	95 ± 4
*Shewanella metallivivens* A14	41.1 ± 0.1	1.3 ± 0.7	857 ± 23	93 ± 1
*Pseudomonas neustonica* A25	59.3 ± 1.0	38.7 ± 0.6	834 ± 15	100 ± 0
*Pseudoalteromonas arctica* A12	41.5 ± 0.9	0.5 ± 0.3	821 ± 21	86 ± 6
*Pseudomonas kurunegalensis* A27	36.1 ± 0.7	0.4 ± 0.1	809 ± 23	99 ± 1
*Marinomonas foliarum* A24	49.6 ± 2.0	16.3 ± 6.1	798 ± 22	100 ± 0
*Shewanella metallivivens* A17	34.3 ± 0.1	0.6 ± 0.2	772 ± 18	85 ± 4
*Shewanella litoralis* A15	38.7 ± 0.2	0.5 ± 0.2	543 ± 15	97 ± 2
*Ectopseudomonas khazarica* Nat2	40.7 ± 0.2	1.0 ± 0.2	377 ± 8	6 ± 3
*Pseudomonas peli* Lo4	42.0 ± 0.5	2.4 ± 0.6	315 ± 9	5 ± 2

## Data Availability

The original sequencing data of the 16S rRNA gene V3–V4 fragments of prokaryotic communities have been deposited at the National Center for Biotechnology Information (NCBI) Sequence Read Archive (SRA; available at http://www.ncbi.nlm.nih.gov/sra/(accessed on 6 July 2026)) under accession number PRJNA1418990. The GenBank/EMBL/DDBJ accession numbers for the 16S rRNA gene sequences of the new bacterial strains are as follows: PX924859, PX924868, PX924912, PX924993, PX925089, PX931340, PX931356, PX931358–PX931360, PX931515, PX931626–PX931628, PX931687, PX931744.

## Supplementary Materials

The following supporting information can be downloaded at https://www.mdpi.com/article/10.3390/microorganisms14081856/s1: Figure S1: Chromatogram of *n*-alkanes in fuel oil maltene fraction; Figure S2: Ion chromatograms showing aromatic compounds distributions in the fuel oil; Figure S3: Matrix of Bray–Curtis’s similarities of studied microbial communities. Color saturations and values in the matrix cells represent the levels of similarity between the corresponding 16S rRNA gene libraries. Values on a diagonal are identically equal to 1.00 and, therefore, are not shown in the matrix; Figure S4: The compositions of microbial communities of seawater (A1, A4, and A8), sand (A2, A5-A7, and A9), and fuel oil (A3) at the level of classes (a), orders (b), and families (c) based on 16S rRNA gene V3-V4 fragments sequences; Figure S5: Linear discriminant analysis effect size (LEfSe) of significant biomarkers diverging the microbial communities of the studied samples. Only taxa with log_10_ (LDA score) > 3.0 are shown; Figure S6: Cladogram of LEfSe significant biomarkers diverging the studied microbial communities of seawater and sand. Colored nodes represent the differential clade abundance depending on the sample type. Only taxa with log_10_ (LDA score) > 3.0 are shown; Figure S7: Functional redundancy analysis of microbial communities based on comparison of 16S rRNA gene libraries of studied samples with the FAPROTAX reference database. Colors of heatmap cells represent proportions of the phylotypes associated with listed ecological functions in corresponding libraries; Figure S8: numbers of aerobic organotrophic bacteria (AOB) and hydrocarbon-oxidizing bacteria (HOB) in seawater samples A1, A4, and A8 (in log_10_(cells number/mL of seawater)), and in sand samples A2, A5, A6, A7, and A9 (in log_10_(cells number/g of sand)). Bacteria incubated at 15 °C were designated as AOB_15 and HOB_15, bacteria incubated at 23 °C were designated as AOB_23 and HOB_23; Figure S9: Chromatograms of the alkane fraction of heavy crude oil in sterile control medium (a) and in media with marine isolates (b–e), together with bar charts showing residual *n*-alkane content in oil (as % relative to *n*-alkane content in the control) degraded by marine isolates *Shewanella* sp. A22 (f), *S. metallivivens* A15 (g), *E. khazarica* Nat2 (h), *P. arctica* A12 (i), and *P. peli* Lo4 (j). Strains were incubated in the medium with heavy crude oil (0.1%, *v*/*v*) at 23 °C for 30 days; Figure S10: Chromatograms of the alkane fraction of fuel oil in sterile control medium (a) and in media with collection bacterial strains (b–d), together with bar charts showing residual *n*-alkane content in fuel oil (as % relative to *n*-alkane content in the control) degraded by strains *P. frederiksbergensis* Ar-K7 (e), *R. erythropolis* TG65 (f), *M. lutaoensis* Pd2 (g), and *P. guguanensis* G3 (h). Strains were incubated in mineral medium with fuel oil (1%, *v*/*v*) at 30 °C for 30 days; Table S1: Macro-component composition of a seawater sample from the Black Sea near Anapa, taken after a fuel oil spill in January 2025; Table S2: Trace element concentrations in the surface waters of the Black Sea and their maximum permissible concentration (MPC) values; Table S3: Group composition of fuel oil samples (in %); Table S4: Peak area ratios of the fuel oil biomarkers for sample 1; Table S5: Number of 16S rRNA gene sequences, observed OTUs and alpha diversity indices in the DNA libraries of studied samples; Table S6: Taxonomic affiliation of bacterial strains isolated from fuel oil contaminated seawater, sand, and fuel oil; Table S7: Taxonomic affiliation of collection hydrocarbon-oxidizing bacterial strains.

## References

[1] McGenity T.J., McKew B.A., Lea-Smith D.J. (2021). Cryptic microbial hydrocarbon cycling. Nat. Microbiol..

[2] Ezra S., Feinstein S., Pelly I., Bauman D., Miloslavsky I. (2000). Weathering of fuel oil spill on the East Mediterranean coast, Ashdod, Israel. Org. Geochem..

[3] Gallego J.R., González-Rojas E., Peláez A.I., Sánchez J., García-Martínez M.J., Ortiz J.E., Torres T., Llamas J.F. (2006). Natural attenuation and bioremediation of Prestige fuel oil along the Atlantic coast of Galicia (Spain). Org. Geochem..

[4] Hazen T.C., Dubinsky E.A., DeSantis T.Z., Andersen G.L., Piceno Y.M., Singh N., Jansson J.K., Probst A., Borglin S.E., Fortney J.L. (2010). Deep-sea oil plume enriches indigenous oil-degrading bacteria. Science.

[5] Wang Q., Zhang S., Li Y., Klassen W. (2011). Potential approaches to improving biodegradation of hydrocarbons for bioremediation of crude oil pollution. J. Environ. Prot..

[6] Atodiresei D., Popa C., Dobref V. (2025). Simulating oil spill evolution and environmental impact with specialized software: A case study for the Black Sea. Sustainability.

[7] Atlas R.M., Hazen T.C. (2011). Oil biodegradation and bioremediation: A tale of the two worst spills in U.S. history. Environ. Sci. Technol..

[8] Camilli R., Reddy C.M., Yoerger D.R., Van Mooy B.A., Jakuba M.V., Kinsey J.C., McIntyre C.P., Sylva S.P., Maloney J.V. (2010). Tracking hydrocarbon plume transport and biodegradation at Deepwater Horizon. Science.

[9] Kimes N.E., Callaghan A.V., Aktas D.F., Smith W.L., Sunner J., Golding B.T., Drozdowska M., Hazen T.C., Suflita J.M., Morris P.J. (2013). Metagenomic analysis and metabolite profiling of deep-sea sediments from the Gulf of Mexico following the Deepwater Horizon oil spill. Front. Microbiol..

[10] Bacosa H.P., Evans M.M., Wang Q., Liu Z.F. (2018). Assessing the role of environmental conditions on the degradation of oil following the Deepwater Horizon oil spill. Oil Spill Environmental Forensics Case Studies.

[11] Godoy-Lozano E.E., Raggi L., Escobar-Zepeda A., Adaya L., Cuervo-Amaya D.H., Gracia A., Sanchez-Flores A., Díaz-Camino C., Pardo-López L. (2026). Enrichment hydrocarbon-degrading bacterial communities from the southern Gulf of Mexico in long-term stored sediments. Biodegradation.

[12] Mason O.U., Hazen T.C., Borglin S., Chain P.S., Dubinsky E.A., Fortney J.L., Han J., Holman H.Y., Hultman J., Lamendella R. (2012). Metagenome, metatranscriptome and single-cell sequencing reveal microbial response to Deepwater Horizon oil spill. ISME J..

[13] Redmond M.C., Valentine D.L. (2012). Natural gas and temperature structured a microbial community response to the *Deepwater Horizon* oil spill. Proc. Natl. Acad. Sci. USA.

[14] Kleindienst S., Grim S., Sogin M., Bracco A., Crespo-Medina M., Joye S.B. (2016). Diverse, rare microbial taxa responded to the Deepwater Horizon deep-sea hydrocarbon plume. ISME J..

[15] Crespo-Medina M., Meile C.D., Hunter K.S., Diercks A.-R., Asper V.L., Orphan V.J., Tavormina P.L., Nigro L.M., Battles J.J., Chanton J.P. (2014). The rise and fall of methanotrophy following a deepwater oil-well blowout. Nat. Geosci..

[16] Wolfe D.A., Hameedi M.J., Galt J.A., Watabayashi D., Short J., O’Clair C., Rice S., Michel J., Payne J.R., Braddock J. (1994). Fate of the oil spilled from the T/V Exxon Valdez in Prince William Sound, Alaska. Environ. Sci. Technol..

[17] Bragg J.R., Prince R.C., Wilkinson J.B., Atlas R. (1992). Bioremediation for Shoreline Cleanup Following the 1989 Alaskan Oil Spill.

[18] Boufadel M.C., Sharifi Y., Van Aken B., Wrenn B.A., Lee K. (2010). Nutrient and oxygen concentrations within the sediments of an Alaskan beach polluted with the Exxon Valdez oil spill. Environ. Sci. Technol..

[19] Shigenaka G. (2014). Twenty-Five Years After the Exxon Valdez Oil Spill: NOAA’s Scientific Support, Monitoring, and Research.

[20] Pritchard P.H., Costa C.F. (1991). EPA’s Alaska oil spill bioremediation project. Environ. Sci. Technol..

[21] Bragg J.R., Prince R.C., Harner E.J., Atlas R.M. (1994). Effectiveness of bioremediation for the Exxon Valdez oil spill. Nature.

[22] Medina-Bellver J.I., Marín P., Delgado A., Rodríguez-Sánchez A., Reyes E., Ramos J.L., Marqués S. (2005). Evidence for in situ crude oil biodegradation after the Prestige oil spill. Environ. Microbiol..

[23] Díez S., Jover E., Bayona J.M., Albaigés J. (2007). Prestige oil spill. III. Fate of a heavy oil in the marine environment. Environ. Sci. Technol..

[24] Lee D., Seo J.M., Kooistra K., Lee H. (2022). Identification of bilge oil with lubricant: Recent oil spill case studies. Environ. Res..

[25] Scarlett A.G., Nelson R.K., Gagnon M.M., Reddy C.M., Grice K. (2024). Very low sulfur fuel oil spilled from the MV Wakashio in 2020 remains in sediments in a Mauritius mangrove ecosystem nearly three years after the grounding. Mar. Pollut. Bull..

[26] Perdigão R., Tomasino M.P., Magalhães C., Carvalho M.F., Almeida C.M.R., Mucha A.P. (2024). Microbial response to a port fuel spill: Community dynamics and potential for bioremediation. Mar. Pollut. Bull..

[27] Alonso-Gutiérrez J., Figueras A., Albaigés J., Jiménez N., Viñas M., Solanas A.M., Novoa B. (2009). Bacterial communities from shoreline environments (Costa da Morte, northwestern Spain) affected by the Prestige oil spill. Appl. Environ. Microbiol..

[28] Matishov G.G., Inzhebeikin Y.I., Savitskii R.M. (2013). The environmental and biotic impact of the oil spill in Kerch Strait in November 2007. Vodn. Resur..

[29] Nemirovskaya I.A., Khaustov A.P., Redina M.M. (2022). Distribution and genesis of hydrocarbons in water and sediments of the Kerch Strait. Geokhimiya.

[30] Nemirovskaya I.A., Zavialov P.O., Khramtsova A.V. (2022). Hydrocarbon pollution in the waters and sediments of the Kerch Strait. Mar. Pollut. Bull..

[31] Soloveva O., Tikhonova E., Zaripova K. (2025). Oil pollution in the Kerch Strait after the “Volgoneft” tanker accident in December 2024. Mar. Biol. J..

[32] Arinina M.P., Gumennyi I.V., Kuzin M.S., Mityukov A.V., Polyakova M.Y., Skvortsov I.Y., Varfolomeeva L.A., Zuev K.V., Malkin A.Y. (2026). Rheological aspects of the fuel oil spill in the Kerch Strait. Mar. Pollut. Bull..

[33] https://world-weather.ru/pogoda/russia/anapa/water-january/.

[34] https://world-weather.ru/pogoda/russia/anapa/water-june/.

[35] (1998). Quantitative Chemical Analysis of Soil. Methods for measuring the mass fraction of petroleum products in mineral, organogenic, organic-mineral soils and bottom sediments using IR spectrometry. Environmental Save Regulation Documents PND F 16.1:2.2.22-98.

[36] Dembicki H. (2017). Practical Petroleum Geochemistry for Exploration and Production.

[37] Semenova E.M., Tourova T.P., Babich T.L., Logvinova E.Y., Sokolova D.S., Loiko N.G., Myazin V.A., Korneykova M.V., Mardanov A.V., Nazina T.N. (2023). Crude oil degradation in temperatures below the freezing point by bacteria from hydrocarbon-contaminated Arctic soils and the genome analysis of *Sphingomonas* sp. AR_OL41. Microorganisms.

[38] Frey B., Rime T., Phillips M., Stierli B., Hajdas I., Widmer F., Hartmann M. (2016). Microbial diversity in European alpine permafrost and active layers. FEMS Microbiol. Ecol..

[39] Chuvochina M., Gerken J., Frentrup M., Sandikci Y., Goldmann R., Freese H.M., Göker M., Sikorski J., Yarza P., Quast C. (2026). SILVA in 2026: A global core biodata resource for rRNA within the DSMZ digital diversity. Nucleic Acids Res..

[40] Pruesse E., Peplies J., Glöckner F.O. (2012). SINA: Accurate high-throughput multiple sequence alignment of ribosomal RNA genes. Bioinformatics.

[41] Rognes T., Flouri T., Nichols B., Quince C., Mahé F. (2016). VSEARCH: A versatile open source tool for metagenomics. PeerJ.

[42] Camacho C., Coulouris G., Avagyan V., Ma N., Papadopoulos J., Bealer K., Madden T.L. (2009). BLAST+: Architecture and applications. BMC Bioinform..

[43] Liu C., Cui Y., Li X., Yao M. (2021). microeco: An R package for data mining in microbial community ecology. FEMS Microbiol. Ecol..

[44] Liu C., Mansoldo F.R., Li H., Vermelho A.B., Zeng R.J., Li X., Yao M. (2025). A workflow for statistical analysis and visualization of microbiome omics data using the R microeco package. Nat. Protoc..

[45] Louca S., Parfrey L.W., Doebeli M. (2016). Decoupling function and taxonomy in the global ocean microbiome. Science.

[46] Louca S., Jacques S.M.S., Pires A.P.F., Leal J.S., Srivastava D.S., Parfrey L.W., Farjalla V.F., Doebeli M. (2017). High taxonomic variability despite stable functional structure across microbial communities. Nat. Ecol. Evol..

[47] Segata N., Izard J., Waldron L., Gevers D., Miropolsky L., Garrett W.S., Huttenhower C. (2011). Metagenomic biomarker discovery and explanation. Genome Biol..

[48] Wickham H. (2016). ggplot2: Elegant Graphics for Data Analysis.

[49] Slowikowski K. (2024). ggrepel: Automatically Position Non-Overlapping Text Labels with ‘ggplot2’. R Package Version 0.9.6. https://cran.r-project.org/web/packages/ggrepel/index.html.

[50] Wei T., Simko V. (2024). corrplot: Visualization of a Correlation Matrix. R Package Version 0.95. https://cran.r-project.org/web/packages/corrplot/index.html.

[51] Hvitfeldt E. (2024). paletteer: Comprehensive Collection of Color Palettes. R Package Version 1.6.0. https://cran.r-project.org/web/packages/paletteer/index.html.

[52] Pebesma E. (2018). Simple features for R: Standardized support for spatial vector data. R J..

[53] Pebesma E., Bivand R. (2023). Spatial Data Science: With Applications in R.

[54] Massicotte P., South A. (2026). rnaturalearth: World Map Data from Natural Earth. R Package Version 1.2.0. https://cran.r-project.org/web/packages/rnaturalearth/index.html.

[55] Oksanen J., Simpson G., Blanchet F., Kindt R., Legendre P., Minchin P., O’Hara R., Solymos P., Stevens M., Szoecs E. (2025). Vegan: Community Ecology Package. R Package Version 2.7-3. https://cran.r-project.org/web/packages/vegan/index.html.

[56] Gursky Y.N. (2003). Geochemistry of the Lithohydrosphere of Inland Seas. Methods of Study and the Processes of Formation of the Chemical Composition of Interstitial Waters in the Bottom Sediments from the Black, Azov, Caspian, Baltic, White and Barents Seas.

[57] Order of the Federal Agency for Fisheries Dated 26 May 2025 No. 296 “On Approval of Water Quality Standards for Water Bodies of Fishery Significance, Including Standards for Maximum Permissible Concentrations of Pollutants in Waters of Water Bodies of Fishery Significance”. http://publication.pravo.gov.ru/document/0001202506020069.

[58] Chuzhikova-Proskurnina O.D., Proskurnin V.Y., Tereshchenko N.N., Kobechinskaya V.G. (2022). Heavy metals in the coastal waters of Russian sector of the Black Sea and the Sea of Azov. Ekosistemy.

[59] Shnyukov E., Yanko-Hombach V. (2020). Mud Volcanoes of the Black Sea Region and Their Environmental Significance.

[60] Yücesoy F., Ergin M. (1992). Heavy-metal geochemistry of surface sediments from the southern Black Sea shelf and upper slope. Chem. Geol..

[61] Kazak E.S., Pozdniakov S.P. (2020). Field study and reactive simulation of iron migration in groundwater during riverbank filtration. Appl. Geochem..

[62] Peters K.E., Walters C.C., Moldowan J.M. (2010). The Biomarker Guide. Volume 1: Biomarkers and Isotopes in the Environment and Human History.

[63] Nemirovskaya I.A., Zavyalov P.O., Medvedeva A.V., Khalikov I.S., Konovalov B.V., Kalgin V.U. (2025). Transformation of fuel oil in the Black Sea two and a half months after the tanker accident. Dokl. Earth Sci..

[64] Zimens M.E., Polovkov N.Y., Zolotareva V.A., Pantserny A.V., Kanatyeva A.Y., Borisov R.S. (2025). Definition of a fuel oil spill source using GC-MS. Pet. Chem..

[65] Howe K.L., Zaugg J., Mason O.U. (2024). Novel, active, and uncultured hydrocarbon-degrading microbes in the ocean. Appl. Environ. Microbiol..

[66] Park S.J., Kim J.G., Jung M.Y., Kim S.J., Cha I.T., Ghai R., Martín-Cuadrado A.B., Rodríguez-Valera F., Rhee S.K. (2012). Draft genome sequence of an ammonia-oxidizing archaeon, “*Candidatus* Nitrosopumilus sediminis” AR2, from Svalbard in the Arctic Circle. J. Bacteriol..

[67] Aylward F.O., Santoro A.E. (2020). Heterotrophic *Thaumarchaea* with small genomes are widespread in the Dark Ocean. mSystems.

[68] Rappé M.S., Connon S.A., Vergin K.L., Giovannoni S.J. (2002). Cultivation of the ubiquitous SAR11 marine bacterioplankton clade. Nature.

[69] Herlemann D.P., Woelk J., Labrenz M., Jürgens K. (2014). Diversity and abundance of “*Pelagibacterales*” (SAR11) in the Baltic Sea salinity gradient. Syst. Appl. Microbiol..

[70] Tucker S.J., Freel K.C., Eren A.M., Rappé M.S. (2025). Habitat-specificity in SAR11 is associated with a few genes under high selection. ISME J..

[71] Giebel H.A., Kalhoefer D., Gahl-Janssen R., Choo Y.J., Lee K., Cho J.C., Tindall B.J., Rhiel E., Beardsley C., Aydogmus Ö.O. (2013). *Planktomarina temperate* gen. nov., sp. nov., belonging to the globally distributed RCA cluster of the marine *Roseobacter* clade, isolated from the German Wadden Sea. Int. J. Syst. Evol. Microbiol..

[72] Giebel H.A., Kalhoefer D., Lemke A., Thole S., Gahl-Janssen R., Simon M., Brinkhoff T. (2011). Distribution of *Roseobacter* RCA and SAR11 lineages in the North Sea and characteristics of an abundant RCA isolate. ISME J..

[73] Zhang C.L., Xie W., Martin-Cuadrado A.B., Rodriguez-Valera F. (2015). Marine Group II Archaea, potentially important players in the global ocean carbon cycle. Front. Microbiol..

[74] Rinke C., Rubino F., Messer L.F., Youssef N., Parks D.H., Chuvochina M., Brown M., Jeffries T., Tyson G.W., Seymour J.R. (2019). A phylogenomic and ecological analysis of the globally abundant Marine Group II archaea (*Ca*. Poseidoniales ord. nov.). ISME J..

[75] Yakimov M.M., Golyshin P.N., Lang S., Moore E.R., Abraham W.R., Lünsdorf H., Timmis K.N. (1998). *Alcanivorax borkumensis* gen. nov., sp. nov., a new, hydrocarbon-degrading and surfactant-producing marine bacterium. Int. J. Syst. Bacteriol..

[76] Yakimov M.M., Giuliano L., Denaro R., Crisafi E., Chernikova T.N., Abraham W.R., Lünsdorf H., Timmis K.N., Golyshin P.N. (2004). *Thalassolituus oleivorans* gen. nov., sp. nov., a marine bacterium confined to the utilization of hydrocarbons. Int. J. Syst. Evol. Microbiol..

[77] Deppe U., Richnow H.H., Michaelis W., Antranikian G. (2005). Degradation of crude oil by an arctic microbial consortium. Extremophiles.

[78] Zhou Y., Wang Y., Yao S., Zhao X., Kong Q., Cui L., Zhang H.J. (2024). Driving mechanisms for the adaptation and degradation of petroleum hydrocarbons by native microbiota from seas prone to oil spills. J. Hazard. Mater..

[79] Harayama S., Kishira H., Kasai Y., Shutsubo K. (1999). Petroleum biodegradation in marine environments. J. Mol. Microbiol. Biotechnol..

[80] Dutta T.K., Harayama S. (2001). Biodegradation of *n*-alkylcycloalkanes and *n*-alkylbenzenes via new pathways in *Alcanivorax* sp. strain MBIC 4326. Appl. Environ. Microbiol..

[81] Hara A., Syutsubo K., Harayama S. (2003). *Alcanivorax* which prevails in oil-contaminated seawater exhibits broad substrate specificity for alkane degradation. Environ. Microbiol..

[82] Röling W.F., Milner M.G., Jones D.M., Fratepietro F., Swannell R.P., Daniel F., Head I.M. (2004). Bacterial community dynamics and hydrocarbon degradation during a field-scale evaluation of bioremediation on a mudflat beach contaminated with buried oil. Appl. Environ. Microbiol..

[83] Liu C., Shao Z. (2005). *Alcanivorax dieselolei* sp. nov., a novel alkane-degrading bacterium isolated from sea water and deep-sea sediment. Int. J. Syst. Evol. Microbiol..

[84] Schneiker S., Martins dos Santos V.A., Bartels D., Bekel T., Brecht M., Buhrmester J., Chernikova T.N., Denaro R., Ferrer M., Gertler C. (2006). Genome sequence of the ubiquitous hydrocarbon-degrading marine bacterium *Alcanivorax borkumensis*. Nat. Biotechnol..

[85] dos Santos V., Sabirova J., Timmis K., Yakimov M., Golyshin P., Timmis K., Mcgenity T., Van Der Meer J., De Lorenzo V. (2010). *Alcanivorax* *borkumensis*. Handbook of Hydrocarbon and Lipid Microbiology.

[86] Head I.M., Jones D.M., Röling W.F. (2006). Marine microorganisms make a meal of oil. Nat. Rev. Microbiol..

[87] Geiselbrecht A.D., Hedlund B.P., Tichi M.A., Staley J.T. (1998). Isolation of marine polycyclic aromatic hydrocarbon (PAH)-degrading *Cycloclasticus* strains from the Gulf of Mexico and comparison of their PAH degradation ability with that of Puget Sound *Cycloclasticus* strains. Appl. Environ. Microbiol..

[88] MacNaughton S.J., Stephen J.R., Venosa A.D., Davis G.A., Chang Y.J., White D.C. (1999). Microbial population changes during bioremediation of an experimental oil spill. Appl. Environ. Microbiol..

[89] Ogino A., Koshikawa H., Nakahara T., Uchiyama H. (2001). Succession of microbial communities during a biostimulation process as evaluated by DGGE and clone library analyses. J. Appl. Microbiol..

[90] Maruyama A., Ishiwata H., Kitamura K., Sunamura M., Fujita T., Matsuo M., Higashihara T. (2003). Dynamics of microbial populations and strong selection for *Cycloclasticus pugetii* following the Nakhodka oil spill. Microb. Ecol..

[91] Meier-Kolthoff J.P., Auch A.F., Klenk H.P., Göker M. (2013). Genome sequence-based species delimitation with confidence intervals and improved distance functions. BMC Bioinform..

[92] Chun J., Oren A., Ventosa A., Christensen H., Arahal D.R., Da Costa M.S., Rooney A.P., Yi H., Xu X.-W., De Meyer S. (2018). Proposed minimal standards for the use of genome data for the taxonomy of prokaryotes. Int. J. Syst. Evol. Microbiol..

[93] Tarhriz V., Nouioui I., Spröer C., Verbarg S., Ebrahimi V., Cortés-Albayay C., Schumann P., Hejazi M.A., Klenk H.P., Hejazi M.S. (2020). *Pseudomonas khazarica* sp. nov., a polycyclic aromatic hydrocarbon-degrading bacterium isolated from Khazar Sea sediments. Antonie Van Leeuwenhoek.

[94] Rudra B., Gupta R.S. (2024). Phylogenomics studies and molecular markers reliably demarcate genus *Pseudomonas sensu stricto* and twelve other *Pseudomonadaceae* species clades representing novel and emended genera. Front. Microbiol..

[95] Jeong D., Baik M.H., Jung E.C., Ko M.S., Um W., Ryu J.H. (2023). Potential of indigenous bacteria driven U(VI) reduction under relevant deep geological repository (DGR) conditions. Environ. Pollut..

[96] Lemaire O.N., Méjean V., Iobbi-Nivol C. (2020). The *Shewanella* genus: Ubiquitous organisms sustaining and preserving aquatic ecosystems. FEMS Microbiol. Rev..

[97] Liu J., Tao L., Zhu D., Zhang S., Wang J. (2026). Mitigating marine oil spills: A review of spill behavior, response technologies, and emerging strategies. Mar. Pollut. Bull..

[98] Scoma A., Yakimov M.M., Daffonchio D., Boon N. (2017). Self-healing capacity of deep-sea ecosystems affected by petroleum hydrocarbons. EMBO Rep..

[99] Wang W., Zhi B., Wang Y., Shao Z. (2025). Maintaining Ocean ecosystem health with hydrocarbonoclastic microbes. ISME Commun..

[100] Kleindienst S., Paul J.H., Joye S.B. (2015). Using dispersants after oil spills: Impacts on the composition and activity of microbial communities. Nat. Rev. Microbiol..

[101] Venosa A.D., Zhu X. (2003). Biodegradation of crude oil contaminating marine shorelines and freshwater wetlands. Spill Sci. Technol. Bull..

[102] Acosta-González A., Martirani-von Abercron S.-M., Rosselló-Móra R., Wittich R.-M., Marqués S. (2015). The effect of oil spills on the bacterial diversity and catabolic function in coastal sediments: A case study on the *Prestige* oil spill. Environ. Sci. Pollut. Res..

[103] Alonso-Gutiérrez J., Costa M.M., Figueras A., Albaigés J., Viñas L., Solanas A.M., Novoa B. (2008). *Alcanivorax* strain detected among the cultured bacterial community from sediments affected by the Prestige oil spill. Mar. Ecol.-Prog. Ser..

[104] Bacosa H.P., Liu Z., Erdner D.L. (2015). Natural sunlight shapes crude oil-degrading bacterial communities in Northern Gulf of Mexico surface waters. Front. Microbiol..

[105] Kostka J.E., Prakash O., Overholt W.A., Green S.J., Freyer G., Canion A., Delgardio J., Norton N., Hazen T.C., Huettel M. (2011). Hydrocarbon-degrading bacteria and the bacterial community response in Gulf of Mexico Beach sands impacted by the deepwater horizon oil spill. Appl. Environ. Microbiol..

[106] Bôto M.L., Magalhães C., Perdigão R., Alexandrino D.A.M., Fernandes J.P., Bernabeu A.M., Ramos S., Carvalho M.F., Semedo M., LaRoche J. (2021). Harnessing the potential of native microbial communities for bioremediation of oil spills in the Iberian Peninsula NW coast. Front. Microbiol..

[107] Sayed K., Baloo L., Sharma N.K. (2021). Bioremediation of Total Petroleum Hydrocarbons (TPH) by bioaugmentation and biostimulation in water with floating oil spill containment booms as bioreactor Basin. Int. J. Environ. Res. Public Health.

[108] Yakimov M.M., Bargiela R., Golyshin P.N. (2022). Calm and frenzy: Marine obligate hydrocarbonoclastic bacteria sustain ocean wellness. Curr. Opin. Biotechnol..

[109] Dede B., Priest T., Bach M., Amann R., Meyerdierks A. (2023). High abundance of hydrocarbon degrading *Alcanivorax* in plumes of hydrothermally active volcanoes in the South Pacific Ocean. ISME J..

[110] Kasai Y., Kishira H., Sasaki T., Syutsubo K., Watanabe K., Harayama S. (2002). Predominant growth of *Alcanivorax* strains in oil-contaminated and nutrient-supplemented sea water. Environ. Microbiol..

[111] Martín-Gil J., Ramos-Sánchez M.C., Martín-Gil F.J. (2004). *Shewanella putrefaciens* in a fuel-in-water emulsion from the Prestige oil spill. Antonie Van Leeuwenhoek.

[112] de Souza Araújo L., Santana L.A.R., Otenio M.H., Nascimento C.W., Cerqueira A.F.L.W., Rodarte M.P. (2024). Biosurfactant production by *Pseudomonas*: A systematic review. Appl. Biochem. Biotechnol..

[113] Henkel M., Geissler M., Weggenmann F., Hausmann R. (2017). Production of microbial biosurfactants: Status quo of rhamnolipid and surfactin towards large-scale production. Biotechnol. J..

[114] Cui J., Hölzl G., Karmainski T., Tiso T., Kubicki S., Thies S., Blank L.M., Jaeger K.E., Dörmann P. (2022). The glycine-glucolipid of *Alcanivorax borkumensis* is resident to the bacterial cell wall. Appl. Environ. Microbiol..

[115] Gharaei S., Ohadi M., Hassanshahian M., Porsheikhali S., Forootanfar H. (2022). Isolation, optimization, and structural characterization of glycolipid biosurfactant produced by marine isolate *Shewanella algae* B12 and evaluation of its antimicrobial and anti-biofilm activity. Appl. Biochem. Biotechnol..

[116] Meng Y., Ou X., Liu Z., Yao S., Hu C., Yu Q., Xia W. (2026). Nanoparticle-assisted structural tailoring of trehalose lipid biosynthesis by *Rhodococcus erythropolis* WJ-2. RSC Adv..

