# Supplementary material for: Microbial Diversity and Hydrocarbon-Oxidizing Bacteria in Coastal Waters and Sands Contaminated by the Fuel Oil Spill in the Black Sea
Source: Microorganisms. 2026 Aug 20;14(8):1856. doi: 10.3390/microorganisms14081856 (PMC13515116; doi:10.3390/microorganisms14081856)
Supplement: Supplementary file 1 [file microorganisms-14-01856-s001.zip › microorganisms-4455830 -Supplementary Materials.pdf]

microorganisms

MDPI

Supplementary Materials

Microbial Diversity and Hydrocarbon-Oxidizing Bacteria in Coastal Waters and Sands Contaminated by the Fuel Oil Spill in the Black Sea

Ekaterina M. Semenova [1], Alexey P. Ershov [1], Tamara L. Babich [1], Diyana S. Sokolova [1], Nataliya G. Loiko [1], Elena A. Bakay [2], Ekaterina S. Kazak [2] and Tamara N. Nazina [1,*]

[1] Winogradsky Institute of Microbiology, Research Center of Biotechnology of the Russian Academy of Sciences, 119071 Moscow, Russia; semenova_inmi@mail.ru (E.M.S.); e.alexey.mail@yandex.ru (A.P.E.); microb101@yandex.ru (T.L.B.); sokolovadiyana@gmail.com (D.S.S.); loikonat@mail.ru (N.G.L.); nazina@inmi.ru (T.N.N.)

[2] Faculty of Geology, Lomonosov Moscow State University, 119234 Moscow, Russian Federation; bakay_lena@inbox.ru (E.A.B.); kanigu@mail.ru (E.S.K.)

* Correspondence: nazina@inmi.ru; Tel.: +7-499-135-0341 (T.N.N.)

This file includes:

Figures S1 to S10

Tables S1 to S7

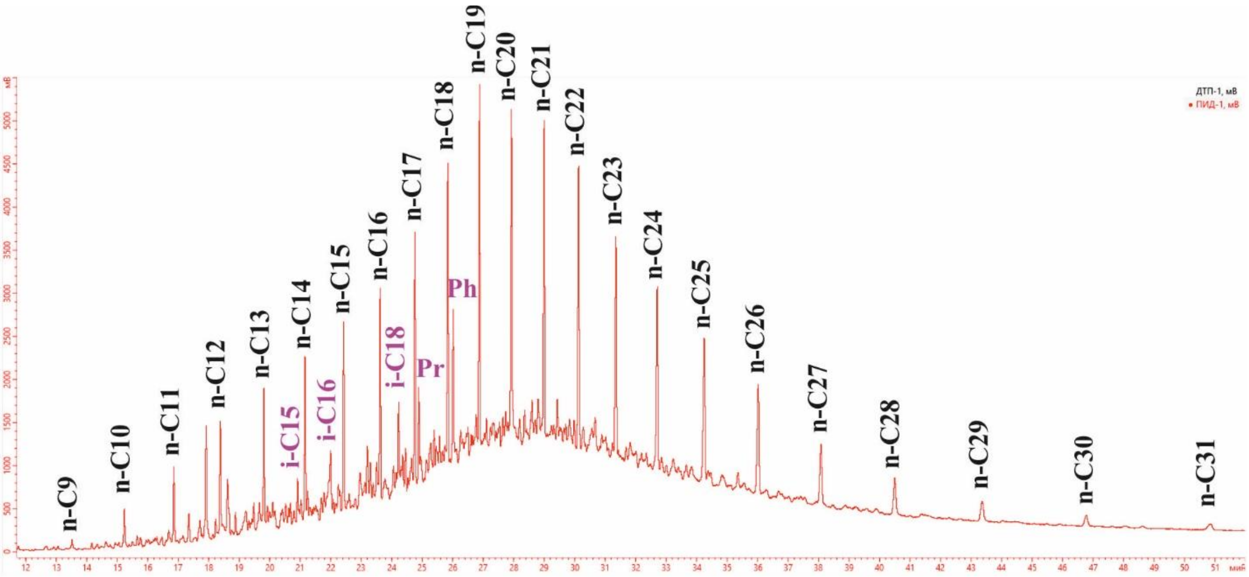

Figure S1. Chromatogram of *n*-alkanes in fuel oil maltene fraction.

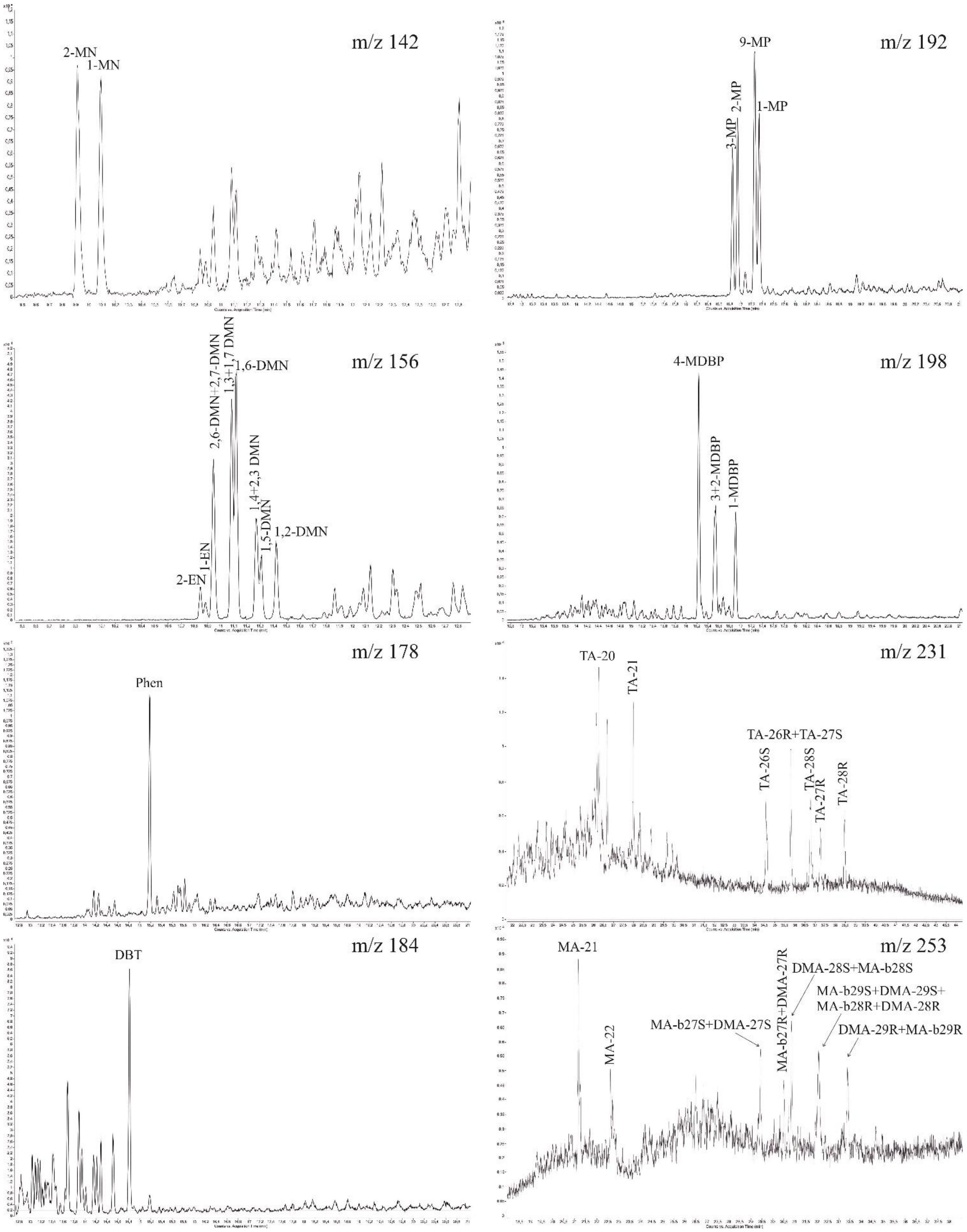

Figure S2. Ion chromatograms showing aromatic compounds distributions in the fuel oil.

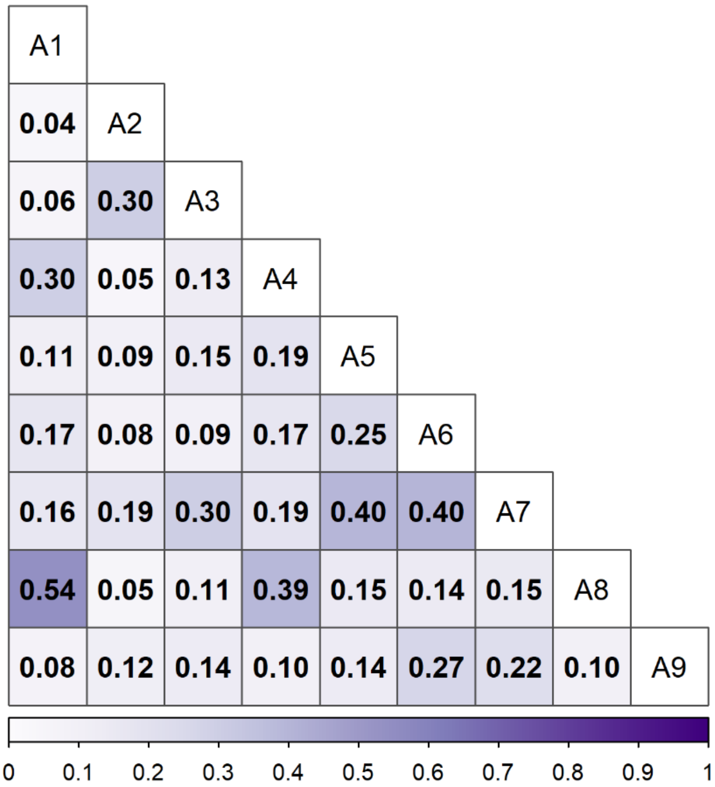

Figure S3 Matrix of Bray-Curtis's similarities of studied microbial communities. Color saturations and values in the matrix cells represent the levels of similarity between the corresponding 16S rRNA gene libraries. Values on a diagonal are identically equal to 1.00 and, therefore, are not shown in the matrix.

Figure S4 (a)

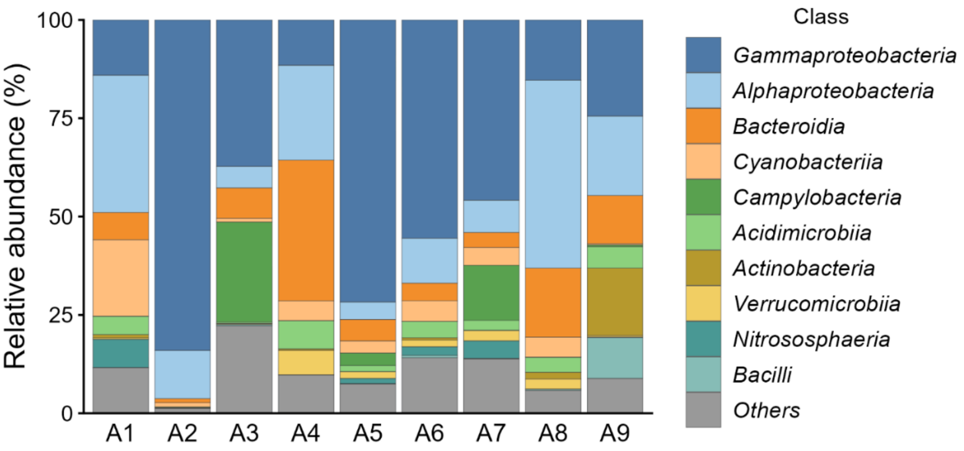

Figure S4 (b)

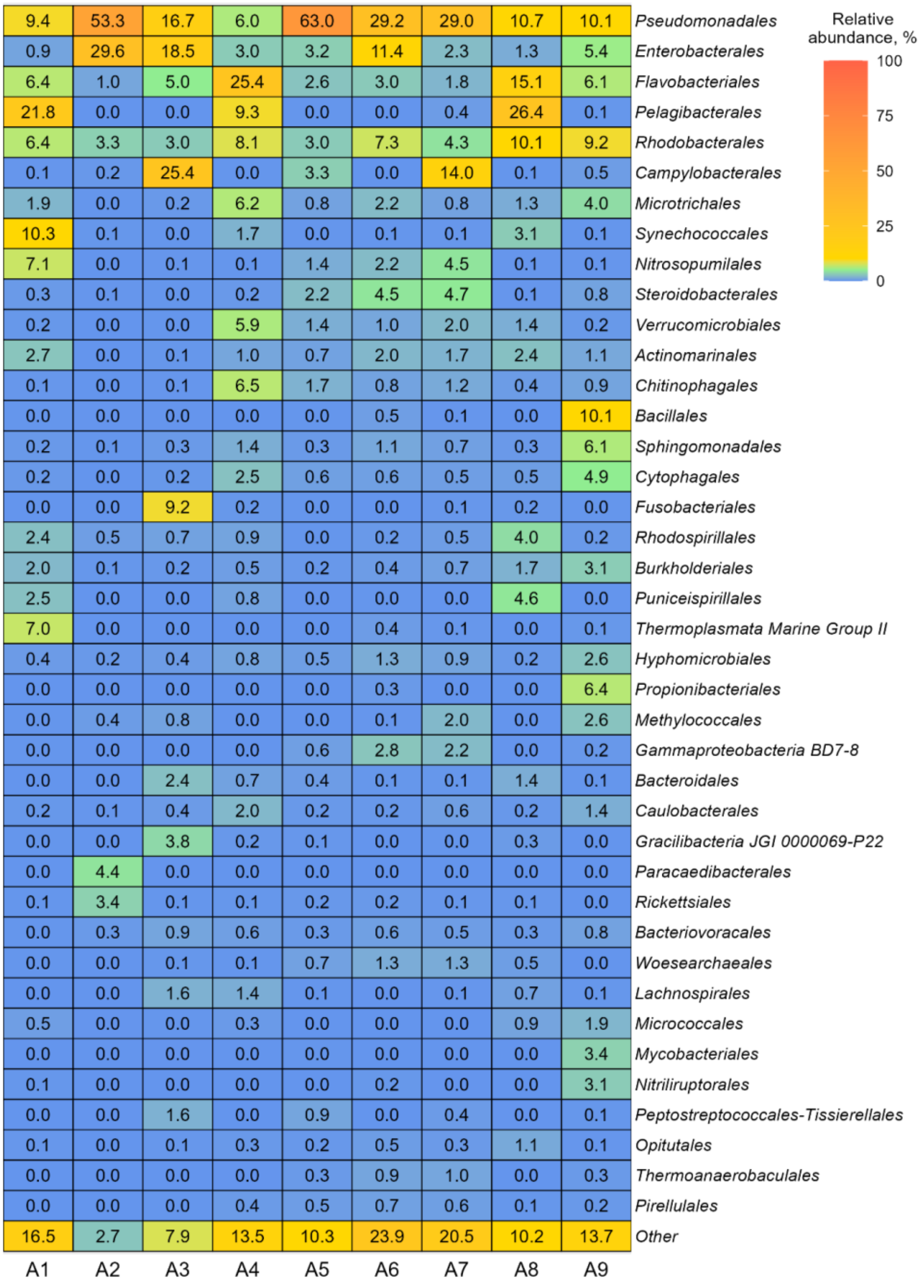

Figure S4 (c)

Family	A1	A2	A3	A4	A5	A6	A7	A8	A9
Oceanospirillaceae	0.0	0.0	0.1	0.0	54.5	0.0	7.2	0.0	0.0
Flavobacteriaceae	3.7	1.0	4.8	25.0	2.3	2.8	1.5	8.9	5.9
Paracoccaceae	6.4	3.3	3.0	8.1	3.0	7.3	4.3	10.1	9.2
Pelagibacterales Clade I	17.4	0.0	0.0	6.6	0.0	0.0	0.1	18.3	0.1
Arcobacteraceae	0.0	0.2	24.6	0.0	3.2	0.0	13.9	0.0	0.4
Saccharospirillaceae	0.1	7.8	7.0	0.2	0.5	0.3	13.0	0.3	2.3
Pseudomonadaceae	0.0	24.9	0.1	0.0	0.1	0.0	2.8	0.0	0.2
Pseudoalteromonadaceae	0.4	15.9	2.7	0.1	0.3	0.7	0.4	0.0	0.2
Moraxellaceae	0.0	8.6	5.9	0.0	0.2	0.1	0.6	0.0	1.7
Cyanobiaceae	10.3	0.1	0.0	1.7	0.0	0.1	0.1	3.1	0.1
Nitrosopumilaceae	7.1	0.0	0.1	0.1	1.4	2.2	4.5	0.1	0.1
Alcanivoracaceae1	0.5	0.5	0.1	0.1	0.5	9.3	0.5	0.1	2.1
Nitrincolaceae	0.0	4.4	1.6	0.0	3.3	2.1	2.1	0.0	0.2
Woeseiaceae	0.3	0.1	0.0	0.2	2.2	4.5	4.7	0.1	0.8
Alteromonadaceae	0.2	1.4	0.6	0.2	0.7	5.1	0.9	0.3	2.9
Pseudomonadales SAR86 clade	5.2	0.0	0.0	1.6	0.0	0.0	0.0	5.5	0.0
Shewanellaceae	0.0	3.2	6.4	0.2	0.1	0.0	0.3	0.1	0.7
Sphingomonadaceae	0.2	0.1	0.3	1.4	0.3	1.1	0.7	0.3	6.1
Vibrionaceae	0.1	0.9	4.3	2.4	0.5	0.0	0.3	0.7	0.1
Fusobacteriaceae	0.0	0.0	8.7	0.2	0.0	0.0	0.1	0.2	0.0
Marinobacteraceae	0.0	0.3	0.0	0.0	0.1	7.3	0.2	0.0	0.9
Saprospiraceae	0.1	0.0	0.1	5.2	1.2	0.6	0.6	0.3	0.6
Pelagibacterales Clade II	2.6	0.0	0.0	1.5	0.0	0.0	0.3	4.1	0.0
Cryomorphaceae	1.1	0.0	0.0	0.3	0.1	0.2	0.1	6.1	0.0
Rubritaleaceae	0.1	0.0	0.0	4.7	0.8	0.4	0.8	1.1	0.0
Puniceispirillales SAR116 clade	2.4	0.0	0.0	0.8	0.0	0.0	0.0	4.6	0.0
Colwelliaceae	0.1	5.7	1.0	0.0	0.8	0.0	0.1	0.0	0.0
Marinomonadaceae	0.0	5.9	1.3	0.0	0.0	0.0	0.1	0.0	0.0
Pseudohongiellaceae	1.3	0.1	0.1	0.1	1.1	2.7	0.5	0.6	0.3
Nocardioidaceae	0.0	0.0	0.0	0.0	0.0	0.3	0.0	0.0	6.4
Rhodospirillales AEGEAN-169 marine group	2.2	0.0	0.0	0.8	0.0	0.0	0.0	3.7	0.0
Ilumatobacteraceae	1.4	0.0	0.1	2.4	0.4	0.5	0.3	1.0	0.5
Planococcaceae	0.0	0.0	0.0	0.0	0.0	0.1	0.0	0.0	6.4
Microtrichaceae	0.5	0.0	0.1	3.7	0.3	0.6	0.4	0.3	0.4
Actinomarinaceae	2.7	0.0	0.0	0.9	0.0	0.0	0.0	2.4	0.0
Cycloclasticaceae	0.0	0.4	0.7	0.0	0.0	0.1	2.0	0.0	2.6
Cellvibrionaceae	0.1	0.2	0.3	1.7	1.9	0.4	0.2	0.6	0.0
Halieaceae	1.1	0.0	0.2	1.5	0.2	0.4	0.4	1.3	0.2
Psychromonadaceae	0.0	1.9	2.7	0.1	0.1	0.0	0.1	0.1	0.0
Hyphomonadaceae	0.2	0.1	0.4	1.9	0.2	0.1	0.6	0.1	1.3
Other	32.2	13.1	22.6	26.2	19.4	50.7	35.3	25.6	47.2

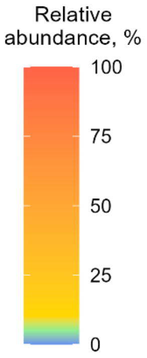

Figure S4. The compositions of microbial communities of seawater (A1, A4, and A8), sand (A2, A5-A7, and A9), and fuel oil (A3) at the level of classes (a), orders (b), and families (c) based on 16S rRNA gene V3-V4 fragments sequences.

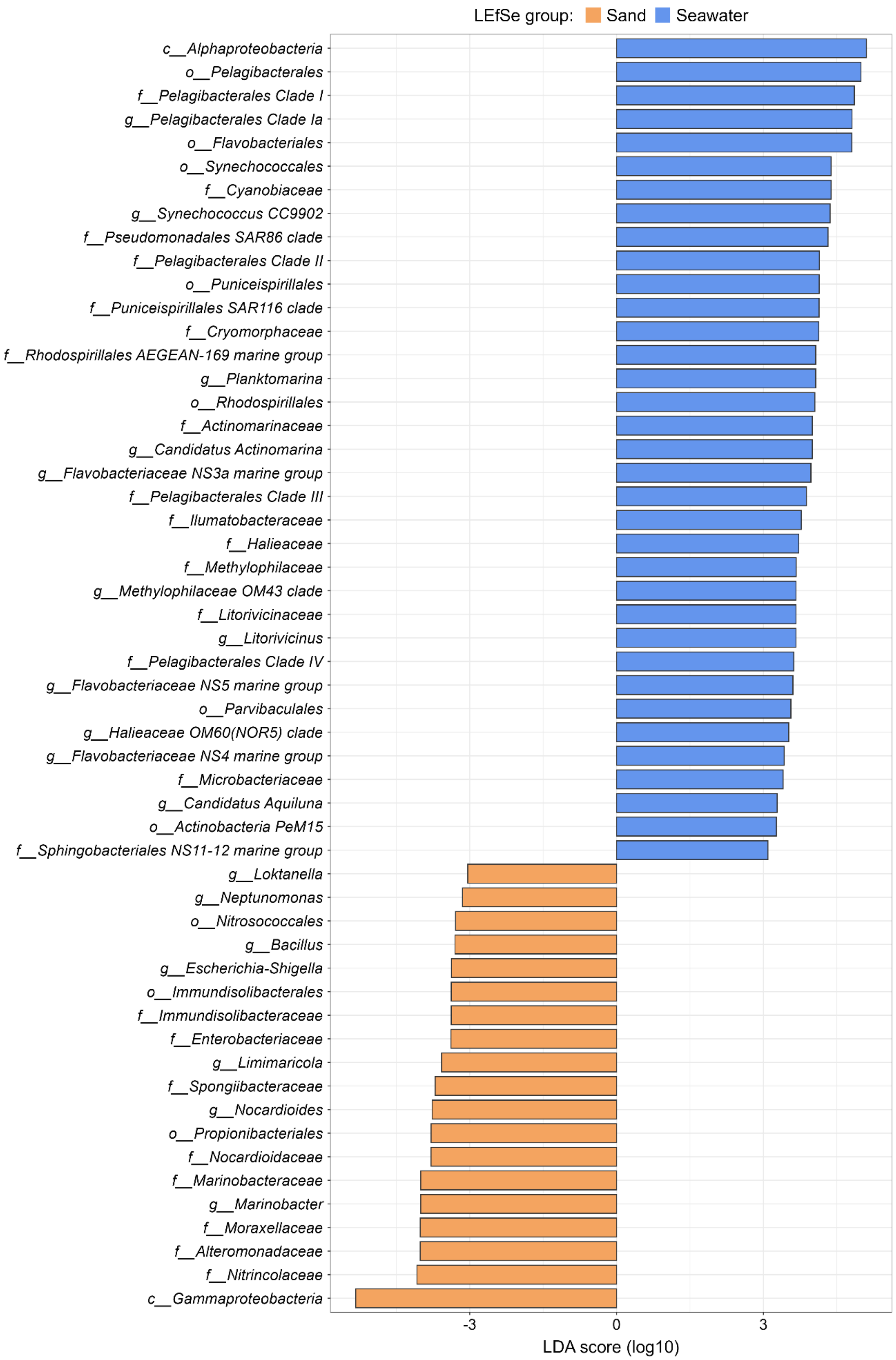

Figure S5. Linear discriminant analysis effect size (LEfSe) of significant biomarkers diverging the microbial communities of the studied samples. Only taxa with $\log_{10}$ (LDA score) > 3.0 are shown.

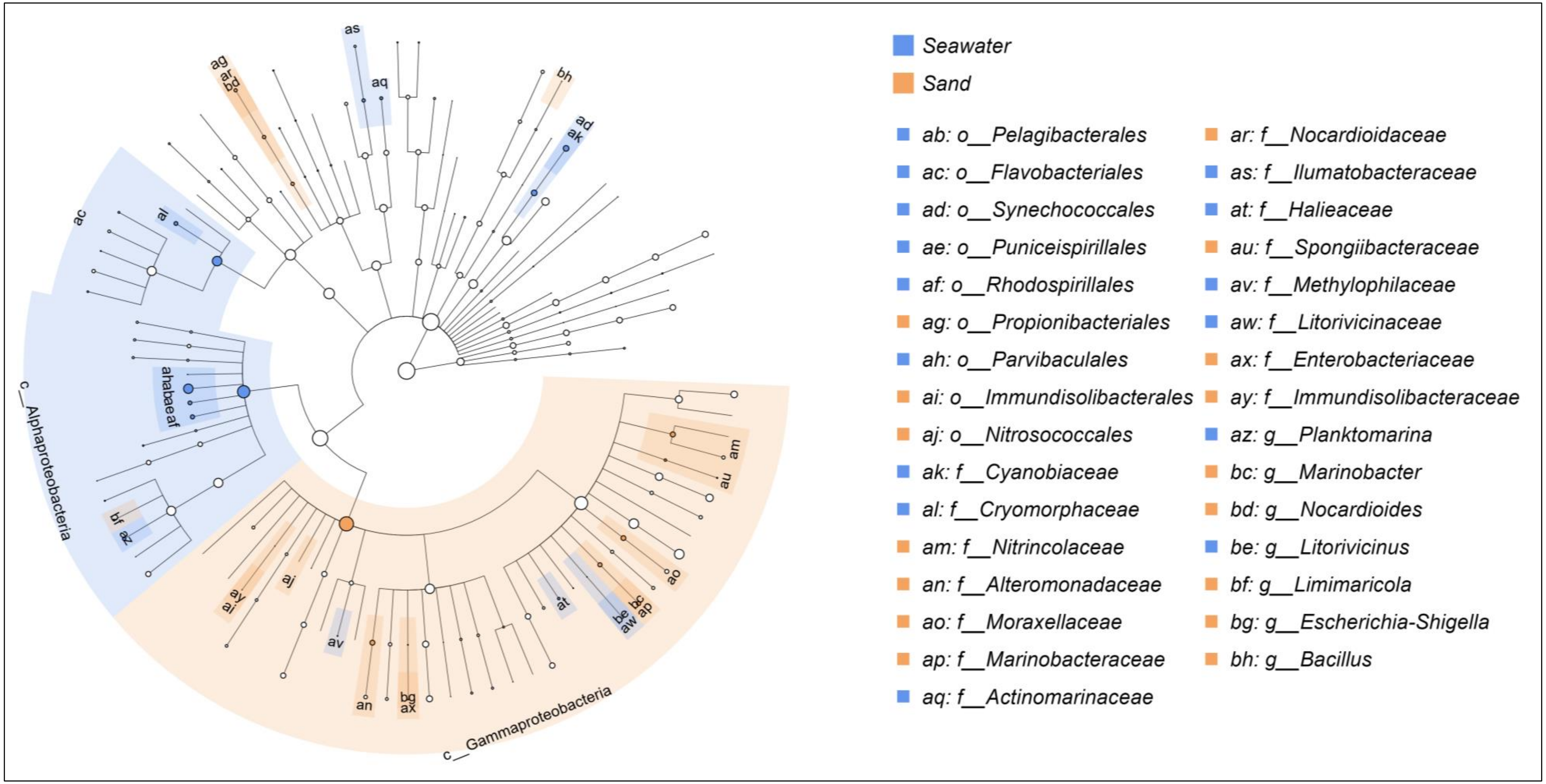

Figure S6. Cladogram of LEfSe significant biomarkers diverging the studied microbial communities of seawater and sand. Colored nodes represent the differential clade abundance depending on the sample type. Only taxa with $\log_{10}$ (LDA score) > 3.0 are shown.

Group	Function	A1	A2	A3	A4	A5	A6	A7	A8	A9
Energy source	photoautotrophy	1.3	2.4	0.6	1.2	1.0	1.1	1.0	1.0	0.6
	anaerobic_chemoheterotrophy	9.7	20.4	17.9	12.2	11.4	12.2	11.0	10.5	9.7
	aerobic_chemoheterotrophy	21.0	15.2	15.8	18.7	16.8	12.9	15.5	19.8	22.2
C-cycle	xylanolysis	0.0	0.5	0.2	0.4	0.0	0.0	0.0	0.0	0.0
	methylotrophy	1.5	1.9	1.5	1.0	0.4	0.4	0.3	1.0	0.8
	methanotrophy	0.9	0.5	0.6	0.2	0.2	0.2	0.2	0.2	0.6
	methanogenesis	0.0	0.0	1.3	0.0	0.2	0.2	0.0	0.2	0.2
	hydrocarbon_degradation	1.3	0.9	0.6	0.2	0.4	0.5	0.3	0.2	1.0
	fermentation	5.2	16.6	11.3	5.0	8.1	6.2	7.3	7.2	5.6
	chitinolysis	0.0	0.5	0.2	0.2	0.0	0.0	0.0	0.0	0.0
	cellulolysis	0.2	0.5	0.2	0.4	0.2	0.2	0.2	0.2	0.5
N-cycle	ureolysis	0.9	0.0	0.0	0.4	0.0	0.2	0.2	0.2	0.3
	nitrogen_respiration	1.3	0.9	0.2	0.4	0.4	0.4	0.5	0.2	0.5
	nitrogen_fixation	0.9	0.5	0.4	0.4	0.0	0.4	0.3	0.2	0.5
	nitrite_respiration	0.2	0.0	0.0	0.0	0.2	0.2	0.2	0.0	0.2
	nitrification	0.9	0.5	0.2	0.2	0.4	0.4	0.2	0.2	0.8
	nitrate_respiration	1.3	0.9	0.2	0.4	0.4	0.4	0.5	0.2	0.5
	nitrate_reduction	2.4	3.8	1.9	1.4	1.0	1.4	1.2	1.0	1.6
	aerobic_nitrite_oxidation	0.0	0.5	0.0	0.0	0.2	0.2	0.0	0.0	0.3
	aerobic_ammonia_oxidation	0.9	0.0	0.2	0.2	0.2	0.2	0.2	0.2	0.5
S-cycle	thiosulfate_respiration	0.2	0.5	0.4	0.2	0.2	0.0	0.2	0.2	0.0
	sulfur_respiration	0.0	0.0	0.6	0.2	0.4	0.2	0.3	0.5	0.3
	sulfite_respiration	0.2	0.0	0.6	0.2	0.2	0.0	0.3	0.2	0.0
	sulfate_respiration	2.6	1.9	7.7	2.9	3.9	4.4	4.9	3.1	3.4
	respiration_of_sulfur_compounds	2.6	2.4	7.9	2.9	3.9	4.4	4.9	3.3	3.5
	dark_sulfide_oxidation	0.2	0.0	0.0	0.0	0.0	0.2	0.0	0.0	0.0
	dark_oxidation_of_sulfur_compounds	0.4	0.5	0.2	0.0	0.0	0.4	0.0	0.2	0.2
Other	reductive_acetogenesis	0.0	0.0	0.0	0.0	0.0	0.0	0.2	0.0	0.2
	predatory_or_exoparasitic	0.0	0.5	0.2	0.2	0.2	0.2	0.2	0.2	0.3
	phototrophy	1.3	2.4	0.6	1.2	1.0	1.1	1.0	1.0	0.6
	photosynthetic_cyanobacteria	1.1	2.4	0.6	1.0	1.0	0.9	0.9	1.0	0.6
	oxygenic_photoautotrophy	1.1	2.4	0.6	1.0	1.0	0.9	0.9	1.0	0.6
	methanol_oxidation	0.6	1.4	0.9	0.8	0.2	0.2	0.2	0.7	0.2
	methanogenesis_using_formate	0.0	0.0	0.2	0.0	0.0	0.0	0.0	0.0	0.0
	methanogenesis_by_disproportionation_of_methyl_groups	0.0	0.0	0.4	0.0	0.0	0.2	0.0	0.2	0.2
	methanogenesis_by_CO2_reduction_with_H2	0.0	0.0	0.6	0.0	0.0	0.2	0.0	0.0	0.0
	manganese_oxidation	0.0	0.0	0.0	0.0	0.4	0.4	0.2	0.2	0.5
	iron_respiration	0.2	0.0	0.2	0.0	0.2	0.2	0.2	0.0	0.2
	intracellular_parasites	0.6	1.4	0.9	1.4	1.2	1.4	1.4	2.1	0.5
	hydrogenotrophic_methanogenesis	0.0	0.0	0.6	0.0	0.0	0.2	0.0	0.0	0.0
	fumarate_respiration	0.2	0.0	0.0	0.0	0.0	0.0	0.2	0.0	0.0
	dark_thiosulfate_oxidation	0.2	0.5	0.2	0.0	0.0	0.2	0.0	0.2	0.2
	dark_iron_oxidation	0.2	0.0	0.0	0.0	0.0	0.0	0.0	0.2	0.0
	dark_hydrogen_oxidation	1.1	0.9	2.3	1.2	1.2	1.2	1.9	1.7	1.4
	aromatic_hydrocarbon_degradation	0.6	0.5	0.2	0.0	0.4	0.4	0.2	0.2	0.3
	aromatic_compound_degradation	0.9	1.9	0.6	0.4	0.8	0.7	0.5	0.5	0.8
	anoxygenic_photoautotrophy_S_oxidizing	0.2	0.0	0.0	0.2	0.0	0.2	0.2	0.0	0.0
	anoxygenic_photoautotrophy	0.2	0.0	0.0	0.2	0.0	0.2	0.2	0.0	0.0
	animal_parasites_or_symbionts	2.4	4.7	2.1	1.2	1.0	0.7	0.9	1.4	1.9
	aliphatic_non_methane_hydrocarbon_degradation	0.2	0.0	0.0	0.0	0.2	0.2	0.0	0.0	0.2

Functional redundancy, % (color scale: 0, 5, 10, 15, 20, 25)

Figure S7. Functional redundancy analysis of microbial communities based on comparison of 16S rRNA gene libraries of studied samples with the FAPROTAX reference database. Colors of heatmap cells represent proportions of the phylotypes associated with listed ecological functions in corresponding libraries.

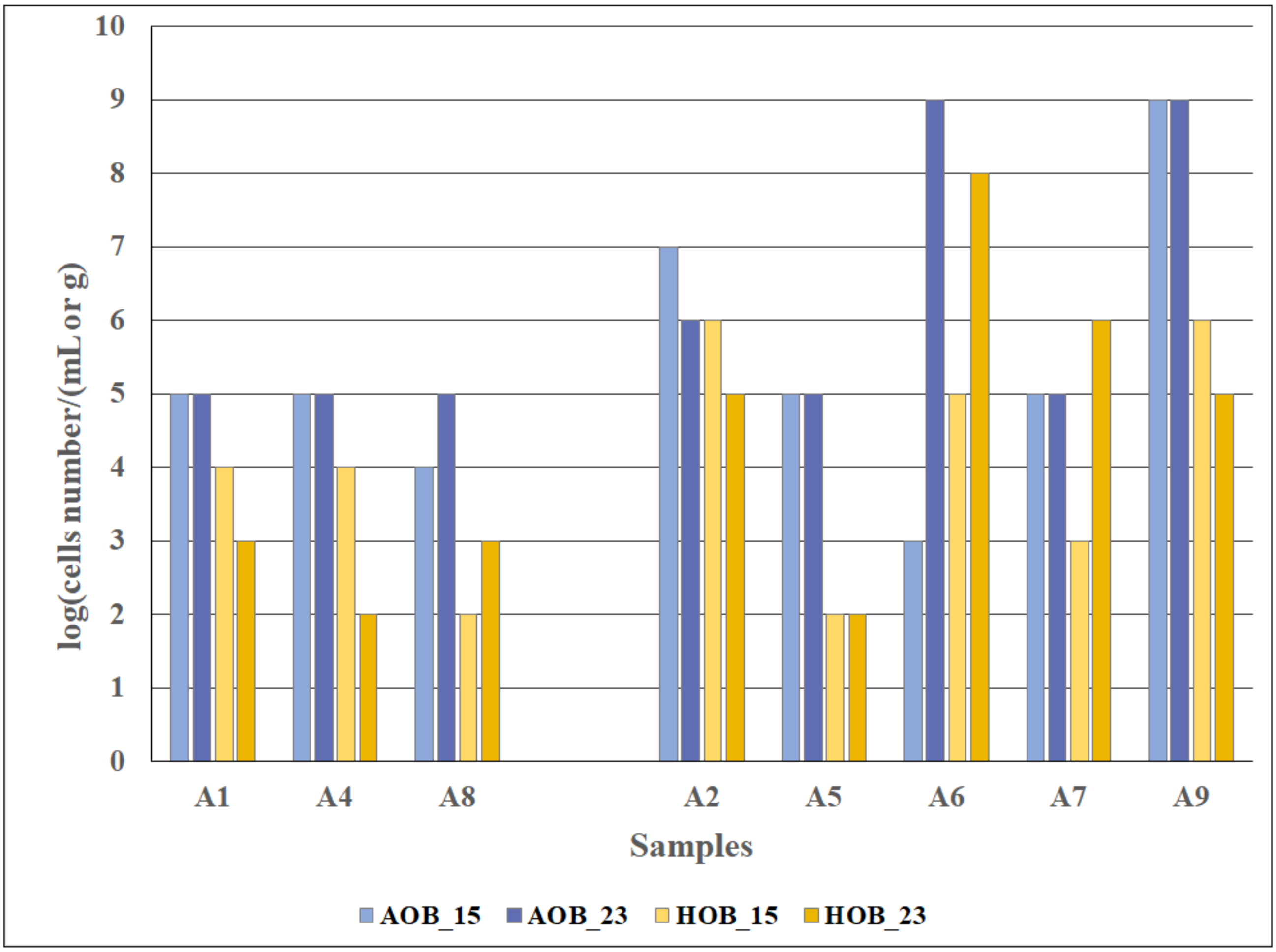

Figure S8. Numbers of aerobic organotrophic bacteria (AOB) and hydrocarbon-oxidizing bacteria (HOB) in seawater samples A1, A4, and A8 (in log_{10}(cells number/mL of seawater)), and in sand samples A2, A5, A6, A7, and A9 (in log_{10}(cells number/g of sand)). Bacteria incubated at 15 °C were designated as AOB_15 and HOB_15, bacteria incubated at 23 °C were designated as AOB_23 and HOB_23.

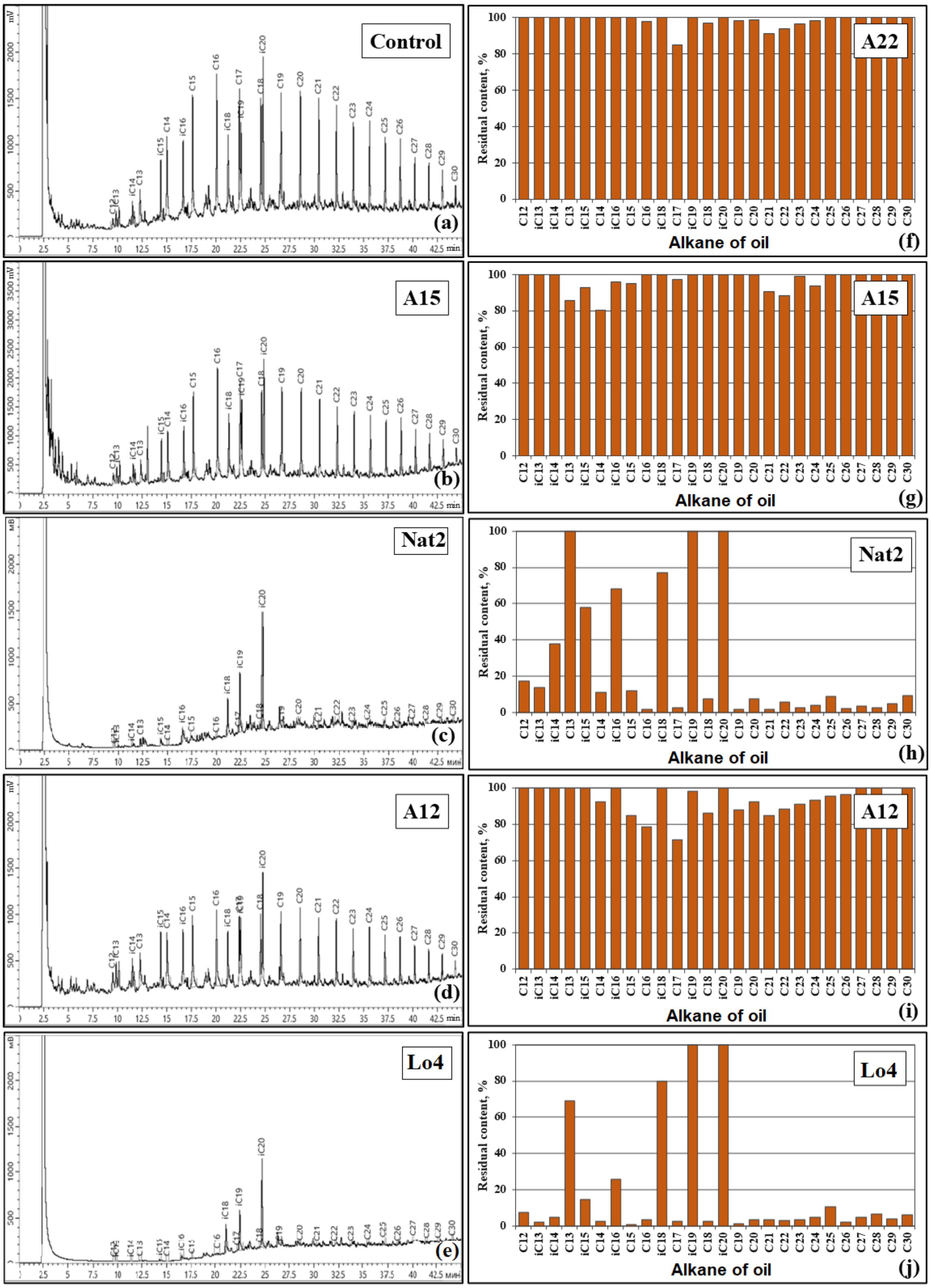

Figure S9. Chromatograms of the alkane fraction of heavy crude oil in sterile control medium (**a**) and in media with marine isolates (**b–e**), together with bar charts showing residual *n*-alkane content in oil (as % relative to *n*-alkane content in the control) degraded by marine isolates *Shewanella* sp. A22 (**f**), *S. metallivivens* A15 (**g**), *E. khazarica* Nat2 (**h**), *P. arctica* A12 (**i**), and *P. peli* Lo4 (**j**). Strains were incubated in the medium with heavy crude oil (0.1%, *v*/*v*) at 23 °C for 30 days.

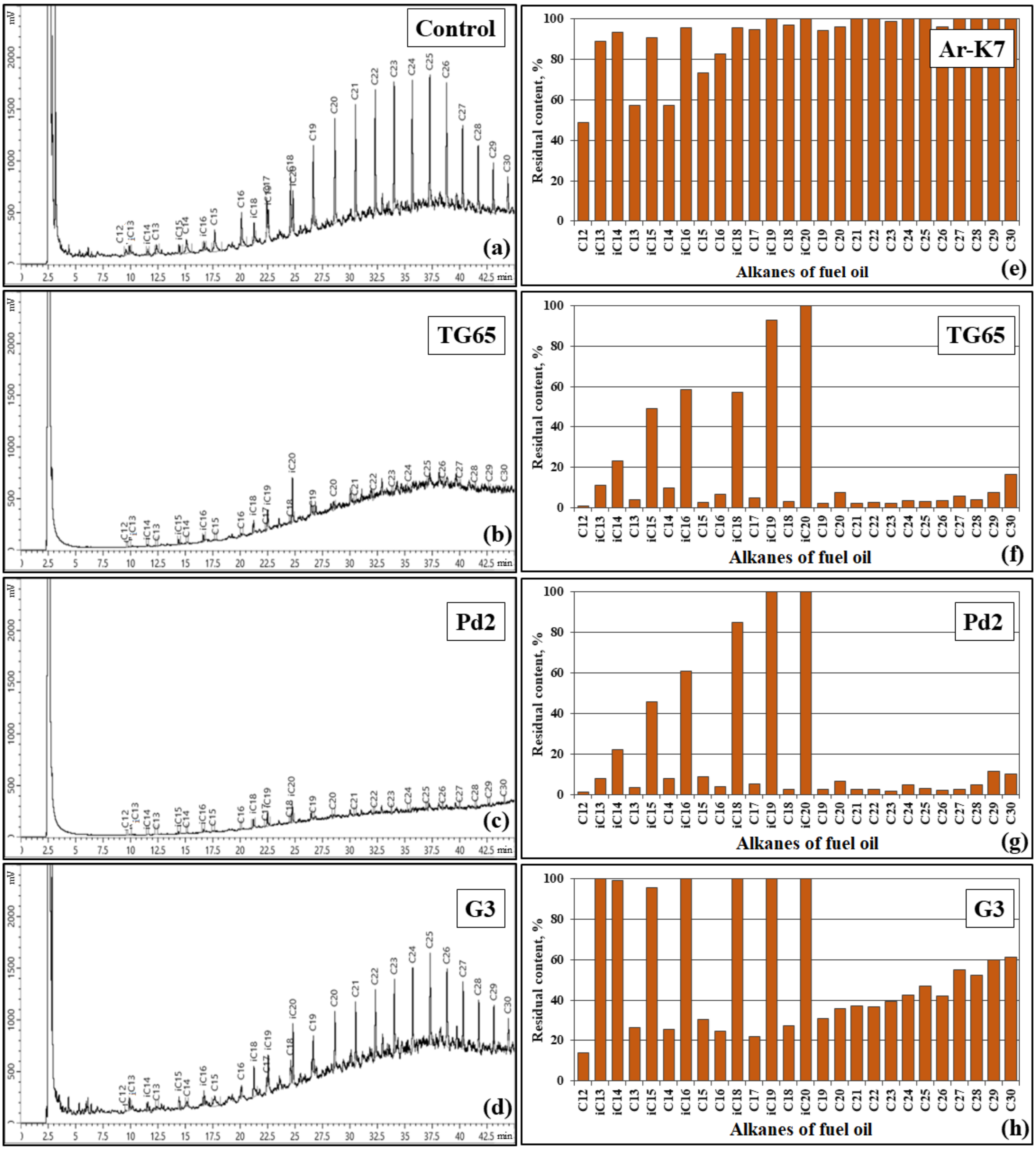

Figure S10. Chromatograms of the alkane fraction of fuel oil in sterile control medium (a) and in media with collection bacterial strains (b–d), together with bar charts showing residual *n*-alkane content in fuel oil (as % relative to *n*-alkane content in the control) degraded by strains *P. frederiksbergensis* Ar-K7 (e), *R. erythropolis* TG65 (f), *M. lutaoensis* Pd2 (g), and *E. guguanensis* G3 (h). Strains were incubated in mineral medium with fuel oil (1%, v/v) at 30 °C for 30 days.

Table S1. Macro-component composition of a seawater sample from the Black Sea near Anapa, taken after a fuel oil spill in January 2025.

Total salinity, g/L	Content[a], mg/L											
	K^+	Na^+	Mg^{2+}	Ca^{2+}	Sr^{2+}	Fe_{total}	Cl^-	SO_4^{2-}	Br^-	B^-	PO_4^{3-}	HCO_3^-
18.46	199.36	5616.6	679.05	230.74	**3.5**	**3.16**	10079.52	1400	37.44	2.98	<0.25	206.45

[a]The content of NH_4^+, NO_2^-, NO_3^-, and F^- was <2 mg/l each.

Table S2. Trace element concentrations in the surface waters of the Black Sea and their maximum permissible concentration (MPC) values.

Parameter	Content, μg/L																			
	Li	Be	Al	Ti	V	Cr	Mn	Co	Ni	Cu	Zn	Rb	Zr	Mo	Cd	Sn	Cs	Ba	W	Pb
MPC	80	0,3	40	60	1	70	10	10	10	1	10	100	70	500	50	112	1000	740	0.8	6
Seawater sample 1	**98.1**	0.01	31.9	11.6	**22.9**	1.92	9.52	0.81	**15.2**	**6.44**	**15.0**	41	0.05	2.98	0.05	0.12	0.05	12.0	0.03	0.35

Table S3. Group composition of fuel oil samples (in %).

Sample No.	Sample description	Volatile HC, water	Oils	Neutral resins	Acidic resins	Asphaltenes
1	Fuel oil from container with water	55	22	6	10	7
2	Fuel oil	56	23	6	9	6
3	Fuel oil with sand	55	24	6	9	6

Table S4. Peak area ratios of the fuel oil biomarkers for sample 1.

Saturated biomarkers	Value	Aromatic biomarkers	Value
Pr/Ph	0.43	TRI/MONO	1.51
Pr/n-C_{17}	0.27	MPI	0.72
Ph/n-C_{18}	0.48	MPI 1	0.72
TRICYC:PENT:STER	21:64:15	MPI 2	0.79
STER C_{27}:C_{28}:C_{29}	40:24:36	4-MDBT/1-MDBT	2.22
DIA/REG	0.93	4-MDBT/DBT	1.86
Ts/Tm	0.21	DBT/Phen	0.73
C_{29}/C_{30} hopane	0.93	4-MDBT/Phen	1.35
Gammacerane/C_{30} hopane	0.18		
hC_{35}/hC_{34}	1.44		

Table S5. Number of 16S rRNA gene sequences, observed OTUs and alpha diversity indices in the DNA libraries of studied samples.

Sample	Number of sequen-ces	Classified sequences	«No relative» sequences	Rejected sequences	Observed OTUs	Chao1 index	Shannon index	Simpson index (1 – D)	Good's coverage
A1	15860	15409	422	29	6960	48188.0	7.25	0.06	0.62
A2	10452	10336	72	44	4260	21645.1	7.05	0.11	0.67
A3	13204	13090	50	64	4805	22041.0	7.13	0.08	0.71
A4	16470	16160	304	6	6537	31496.6	7.66	0.02	0.69
A5	12137	12037	87	13	3356	11341.7	5.49	0.30	0.80
A6	13858	13777	74	7	5194	16334.6	7.44	0.03	0.73
A7	10429	10314	110	5	4219	14687.9	7.17	0.05	0.70
A8	13865	13752	112	1	4281	20174.6	6.69	0.05	0.76
A9	12028	12004	21	3	4098	12408.2	7.14	0.02	0.76

Table S6. Taxonomic affiliation of bacterial strains isolated from fuel oil contaminated seawater, sand, and fuel oil.

Class, family, strain	Closely related type strain, 16S rRNA gene acc. number	16S rRNA gene similarity, %	Accession number in GenBank	T[a], °C	Source of isolation[b]
1	2	3	4	5	6
Gammaproteobacteria					
Shewanellaceae					
A11	*Shewanella vesiculosa* M7, NR_042710.1	100.0	PX924859	15	SW
A14	*Shewanella metallivivens* ER-Te-42B-Ligh, MZ905343.1	98.9	PX924912	15	SW
A15	*Shewanella litoralis* MMS16-UL482, KX621128.1	98.8	PX931687	23	SW
A17	*Shewanella metallivivens* ER-Te-42B-Ligh, MZ905343.1	99.1	PX925089	23	SW
A22	*Shewanella litoralis* MMS16-UL482, KX621128.1	98.8	PX931340	15	S
Pseudoalteromonadaceae					
A12	*Pseudoalteromonas arctica* A 37-1-2, OQ626014.1	100.0	PX924868	15	SW
Pseudomonadaceae					
A16	*Halopseudomonas gallaeciensis* V113, NR_178382.1	100.0	PX924993	23	SW
A25	*Pseudomonas neustonica* PAMC 28426, NR_178930.1	99.9	PX931744	15	S
A27	*Pseudomonas kurunegalensis* RW1P2, NR_178288.1	100.0	PX931358	23	S
Nat5	*Pseudomonas aeruginosa* B764/15, OQ613252.1	100.0	PX931515	23	FO
Lo4	*Pseudomonas peli* R-20805, NR_042451.1	99.8	PX931528	23	S
Nat2	*Ectopseudomonas khazarica* TBZ2, NR_169334.1	99.9	PX931359	23	FO
Lo5	*Ectopseudomonas khazarica* TBZ2, NR_169334.1	100.0	PX931627	23	S
Alcanivoracaceae					
Nat3	*Alloalcanivorax* (previously *Alcanivorax*) *dieselolei* B-5, NR_074734.1	99.0	Nd[c]	23	FO
Oceanospirillaceae					
A24	*Marinomonas foliarum* IVIA-Po-155, EU188444.1	99.0	PX931356	15	S

Table S6. Cont.

1	2	3	4	5	6
Vibrionaceae					
A26	*Vibrio diazotrophicus* NS1, NR_026123.1	100.0	PX931360	23	S
Alphaproteobacteria					
Phyllobacteriaceae					
FW	*Nitratireductor aquibiodomus* NL21, NR_025262.1	100.0	PX931626	23	S
Cytophagia					
Cyclobacteriaceae					
Lo2	*Echinicola sediminis* strain 001-Na2, NR_158015	100	Nd	23	FO

Note: [a], T, temperature of isolation; [b], Source of isolation: SW, seawater; S, sand; FO, fuel oil; [c], Nd, not deposited in this study for that specific strain.

Table S7. Taxonomic affiliation of collection hydrocarbon-oxidizing bacterial strains.

Strain	Closest type strain, 16S rRNA gene acc. number	16S rRNA gene similarity, %	Accession number in GeneBank	Temperature range (optimum), °C	NaCl range (optimum), % (w/v)
Ar-K10	*Arthrobacter alpinus* S6-3, NR_117254	100	MZ411481	–1.5…25 (5–9)	0–8.0 (2.0–4.0)
M3-1	*Aeromonas salmonicida* subsp. *pectinolytica* 34mel, CP022426.1	99.7	MW853692	5–42 (15)	0–7.5 (0–1.0)
Pd1	*Marinobacter lutaoensis* T5054, NR_025116.1	99.9	PX945890	20–50 (37)	1–16 (3–5)
Pd2	*Marinobacter lutaoensis* T5054, NR_025116.1	100	PX945895	20–50 (37)	1–16 (3–5)
M6-6	*Pseudomonas brenneri* BD11-00181, KU647658	99.9	MW853771	5–35 (10–15)	0–>7.5 (0.5)
Ar-K7	*Pseudomonas frederiksbergensis* DSM 13022, NR_117177.1	98.9	MZ411479	–1.5 25 (5–9)	0–1.5 (0.2)
G3	*Ectopseudomonas guguanensis* CC-G9A, NR_135725.1	99.9	PX970282	5–47 (15)	0–6.0 (0.5–3.0)
M11-25	*Pseudomonas kielensis* MBT-1, NR_181570.1	99.9	OM273845	5–35 (15)	0–6.0 (0–1.0)
TG21	*Pseudomonas kielensis* MBT-1, NR_181570.1	100	PX946129	5–37 (15)	0–4.5 (1.5)
M22-22H	*Pseudomonas yamanorum* 8H1, NR_178342.1	100	PX457726	5–37 (15)	0–8.0 (0.5–2.0)
HO-KS22	*Rhodococcus erythropolis* DSM 43066, KJ476725.1	99.8	MN622878	5–37 (28)	0–12.0 (7.0)
TG65	*Rhodococcus erythropolis* JCM 3201, AB429553.1	100	PX946182	5–37 (30)	0–7.0 (3.0–4.0)
TG82	*Rhodococcus erythropolis* JCM 3201, AB429553.1	100	PX946185	5–37 (15)	0–6.0 (0–1.0)
